# Informative relational learning for adverse reaction prediction with enhanced generalization to novel drugs

**DOI:** 10.1093/bioinformatics/btag494

**Published:** 2026-07-02

**Authors:** Shuge Sun, Dalin Zhang, Hongjun Chu, Xinyi Gong

**Affiliations:** Space Information Research Institute, Hangzhou Dianzi University, Baiyang, Hangzhou, Zhejiang 310018, China; Space Information Research Institute, Hangzhou Dianzi University, Baiyang, Hangzhou, Zhejiang 310018, China; Space Information Research Institute, Hangzhou Dianzi University, Baiyang, Hangzhou, Zhejiang 310018, China; Space Information Research Institute, Hangzhou Dianzi University, Baiyang, Hangzhou, Zhejiang 310018, China

## Abstract

**Motivation:**

Accurate prediction of adverse drug reactions (ADRs) is essential for drug safety surveillance, and recent advances in machine learning with heterogeneous biomedical information have improved predictive performance. However, two challenges remain: current methods often learn inadequate ADR representations that fail to capture dependencies among ADRs, and generalize poorly to novel drugs.

**Results:**

To obtain informative ADR embeddings, we construct a multi-source, multi-relational ADR graph that integrates hierarchical structure and empirical ADR co-occurrence, and apply a relational graph convolutional network (R-GCN) to learn relation-aware ADR representations. To enhance generalization to novel drugs, we exploit the hierarchical structure of the Anatomical Therapeutic Chemical (ATC) classification to link drugs via shared higher-level categories for effective knowledge transfer and model these relations with an R-GCN. We further introduce a Conditional Domain Adversarial Network (CDAN) to reduce distribution shifts between known and novel drugs by aligning features conditioned on predicted ADR labels, learning domain-invariant yet task-relevant representations. Additionally, to exploit similar ADR patterns among related drugs, we introduce a dual-branch mixture-of-experts (Dual-MoE) module where each expert captures ADR commonalities within a drug category in one branch, while a separate branch models global patterns. Extensive experiments show that our method consistently outperforms seven baselines, achieving F1 improvements of 4.3% and 4.7% over the best baseline on two datasets, respectively, with more balanced precision–recall trade-offs. It also improves AUC on uncommon ADRs by 7% more than on common ADRs, and remains more robust under data sparsity, with more gradual performance degradation as training data decreases.

**Availability and implementation:**

The code of our model is available at https://github.com/fzsdb/Knowledge-guided-ADR-prediction.git.

## 1 Introduction

Adverse drug reactions (ADRs) are harmful and unintended responses to drugs and account for a substantial proportion of hospital admissions, leading to increased morbidity and reduced quality of life (Tongaonkar *et al.* 2023, Komagamine 2024). These impacts highlight the importance of early ADR risk identification (Dey *et al.* 2018). Beyond patient safety, ADR also affects drug development, as many promising compounds are discontinued due to safety concerns despite years of research and substantial investment (Quan *et al.* 2013, DiMasi *et al.* 2016, Sun *et al.* 2022). Therefore, accurate ADR prediction is crucial for both patient protection and reducing attrition in drug development pipelines.

Traditional ADR identification approaches, including in vitro assays, animal studies, and post-marketing surveillance systems such as the FDA Adverse Event Reporting System (FAERS) (Sakaeda *et al.* 2011), face well-known limitations, including high costs, slow response, and underreporting bias (Berlin *et al.* 2008, Bailey *et al.* 2014, Lavertu *et al.* 2021). To address these limitations and enable earlier risk assessment, computational ADR prediction methods have increasingly leveraged advances in machine learning, integrating diverse information to improve predictive performance and interpretability (Zhuang *et al.* 2023, Farnoush *et al.* 2024, Li *et al.* 2024b).

Despite recent advances, existing methods still exhibit notable limitations in ADR representation and relation modeling. Many approaches do not explicitly learn ADR representations, instead predicting ADRs solely from drug-centric features (Luo *et al.* 2023, Gao *et al.* 2024, Tian *et al.* 2025). More advanced methods construct ADR representations using predefined knowledge (Zhao *et al.* 2023), such as encoding ADRs based on associated genes (Li *et al.* 2024a). Although informative, these representations fail to capture real-world ADR co-occurrence patterns (i.e. ADRs that frequently occur together within the same drug) across multiple drugs. Ignoring such information overlooks meaningful empirical dependencies, as ADR co-occurrence reflects how side effects manifest jointly under specific dosages, patient populations, and treatment conditions, often revealing relationships absent from curated knowledge bases or not yet formally documented (Alomar 2014). In addition, some methods adopt knowledge graph-based approaches to model ADRs and their relations with drugs and other biomedical entities, such as drug targets (Zhang *et al.* 2021, Li *et al.* 2024b). Nevertheless, these approaches face a critical limitation: uncommon ADRs are typically associated with only a few drugs that are simultaneously linked to numerous frequent ADRs and other biological entities, causing their subtle yet distinctive signals to be overwhelmed by dominant information.

In addition to limitations in ADR representation, current methods often exhibit lower performance when predicting ADRs for novel drugs (Zhao *et al.* 2023, Li *et al.* 2024a, 2025). ADR prediction often relies on referencing drugs with similar properties (e.g. indications and targets), based on the assumption that similar drugs tend to exhibit similar ADRs (Winnenburg *et al.* 2015, Zhai *et al.* 2025). However, for novel drugs, the relational links between their uncommon properties and known drugs may be sparse, making it difficult for models to transfer knowledge from existing drugs to unseen ones. Current approaches lack strategies to handle out-of-distribution (OOD) drugs in novel-drug scenarios. In these settings, test drugs are not only unseen during training but may also be dissimilar to training drugs, for example, by exhibiting chemical scaffolds that have not been observed before, under which representations learned from known drugs often fail to generalize (Ji *et al.* 2022, Tossou *et al.* 2024).

**Figure 1:**
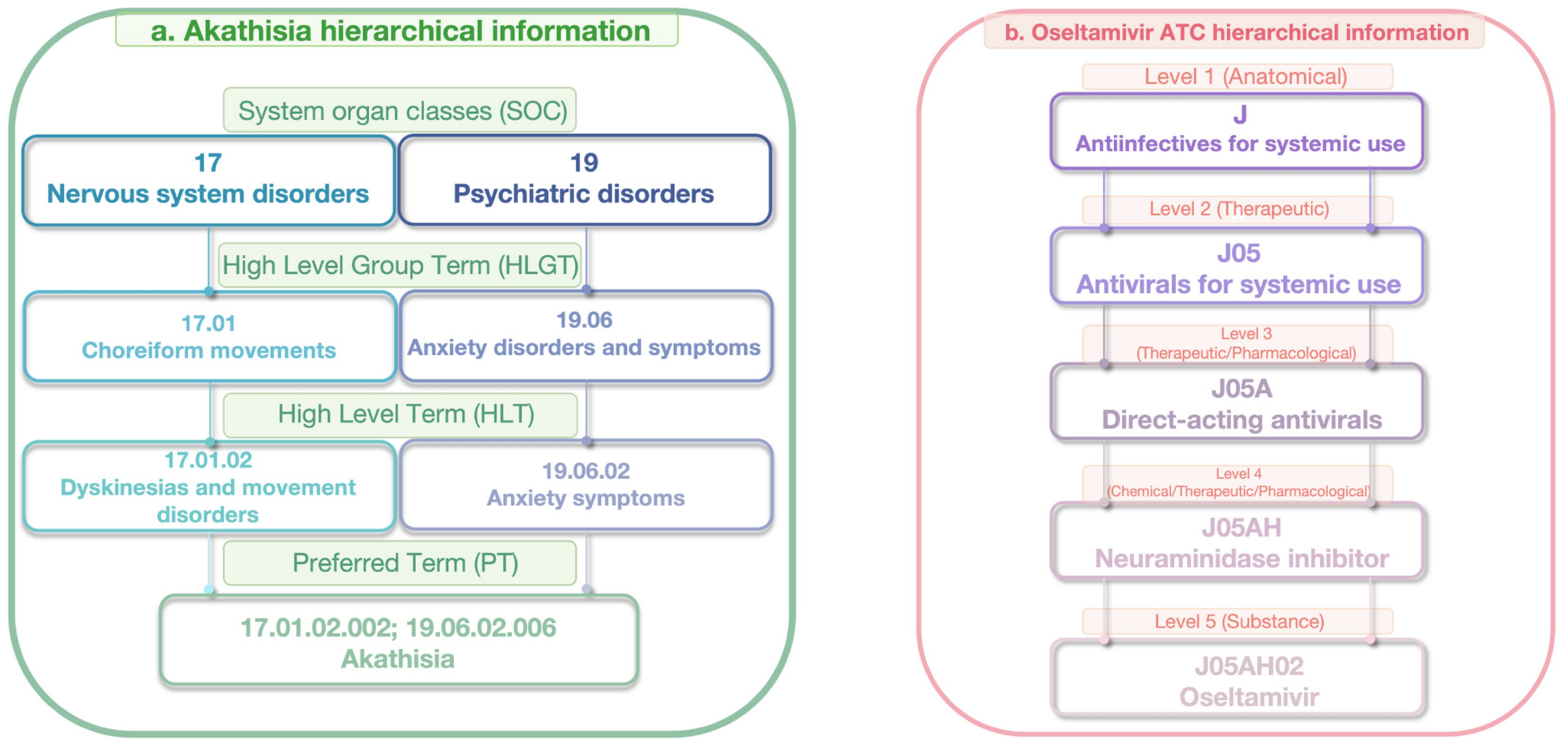
Hierarchical representation of ADR Akathisia and drug Oseltamivir. (a) Akathisia hierarchical information in the ADReCS database. Each ADR term is associated with one or more ADReCS IDs (xx.xx.xx.xxx), Akathisia has two ADReCS IDs: 17.01.02.002 (Nervous system) and 19.06.02.006 (Psychiatric disorders). (b) The ATC hierarchical classification of Oseltamivir comprises five levels, which progressively refine from general antiviral agents to the specific substance Oseltamivir.

To mitigate the limitations in ADR representation, we propose to build a multi-source, multi-relational graph that integrates predefined hierarchical structures and empirical co-occurrence patterns to capture informative ADR associations. The hierarchical structure is defined by ADReCS (Adverse Drug Reaction Classification System) (Cai *et al.* 2015), a standardized ontology for adverse drug reactions. Figure 1(a) illustrates this hierarchy using the ADR Akathisia as an example. ADRs are organized from system organ class (SOC; see Table S1, available as supplementary data at *Bioinformatics* online for more information) to specific reactions, where related ADRs share common ancestors at different levels. This hierarchy enables broader categories to convey shared semantic properties while deeper levels capture more specific distinctions Wang *et al.* (2025). Moreover, the hierarchy anchors rare ADRs within a semantically rich medical taxonomy, capturing intraclass homogeneity and enabling more stable and noise-resilient representations. Beyond predefined hierarchies, we also connect ADRs through real-world co-occurrence patterns to capture their statistical dependencies. We then construct an ADR knowledge graph integrating these relations and employ an R-GCN for multi-relational propagation, enabling hierarchically related or frequently co-occurring ADRs to exert stronger mutual influence and produce fine-grained embeddings.

Limited generalization to novel drugs is mitigated by first establishing more robust connections between novel drugs and known drugs. Specifically, we introduce the Anatomical Therapeutic Chemical (ATC) classification system de la Salud (1996), with R-GCN-based hierarchical modeling. ATC is a five-level hierarchy maintained by the World Health Organization that organizes drugs by the organ or system they act on and their therapeutic, pharmacological, and chemical properties, with the lowest level corresponding to a specific active chemical substance (i.e. a drug in this task). An illustration of this hierarchy for oseltamivir is shown in Fig. 1b, with additional ATC information provided in Table S2 and S3, available as supplementary data at *Bioinformatics* online. Novel drugs may have uncommon fine-grained properties that hinder direct connections to known drugs. However, within the ATC hierarchy, these properties still fall under broader pharmacological, therapeutic, or organ system categories, where shared higher-level classes create coarser yet meaningful links that anchor novel drugs within the relational structure. Additionally, we incorporate co-occurrence relations between ATC nodes (i.e. categories frequently associated with the same ADRs), allowing novel drugs to inherit empirical patterns from neighboring nodes. Modeling with R-GCN enables drugs sharing more hierarchical ancestors to be embedded closer, facilitating fine-grained transfer of ADR knowledge from related known drugs.

To mitigate OOD-related novel-drug generalization issues, we employ Conditional Domain Adversarial Networks (CDANs) (Long *et al.* 2018) to learn domain-invariant features. CDAN aligns feature distributions between training and test samples through adversarial learning while conditioning on label information to preserve discriminative signals, thereby improving generalization to novel drugs.

Finally, motivated by the tendency of similar drugs to share ADR patterns, we explicitly cluster drugs and model each cluster with a specialized sub-module to better capture class-specific patterns. We directly leverage the ATC classification as a natural clustering criterion and adopt a mixture-of-experts framework, assigning one intra-class expert per cluster to model characteristic ADR patterns. Additionally, shared inter-class experts capture cross-category dependencies, forming a Dual-MoE that jointly models within- and cross-category relations.

Experimental results demonstrate that the proposed model consistently outperforms all baseline methods, exceeding the strongest baseline by 4.7% on ADReCS and 4.3% on SIDER, while exhibiting a more balanced precision–recall trade-off and a stronger ability to detect positive ADR cases. Comprehensive ablation studies validate the contributions of each core component, while robustness analyses under data sparsity confirm model stability, and ADR frequency stratification highlights its effectiveness on infrequent ADRs.

## 2 Materials and methods

### 2.1 Problem definition

We formulate ADR prediction as a binary classification problem over drug–ADR pairs. Let D denote the set of drugs and R denote the set of ADRs. For a drug $d_{i}\in D$ and an ADR $r_{j}\in R$, each sample corresponds to a pair $\left(d_{i},r_{j}\right)$ with label $y_{ij}\in \{0,1\}$, where $y_{ij}=1$ indicates that an association between $d_{i}$ and $r_{j}$ has been reported, and $y_{ij}=0$ otherwise. Let $P_{\text{train}}$ denote the set of drug–ADR pairs in the training data, and let $P_{\text{test}}$ denote the set of pairs evaluated during testing. To assess model generalization under different practical scenarios, we evaluate performance under two settings:

#### 2.1.1 Novel Drug Setting (NDS)

The goal is to predict ADRs for drugs that are not observed during training. In this setting, the drug sets of the training and test data are disjoint, while the ADR set remains unchanged:


$$D_{\text{test}}^{\left(\text{NDS}\right)}\cap D_{\text{train}}^{\left(\text{NDS}\right)}=\emptyset ,\;R_{\text{test}}^{\left(\text{NDS}\right)}=R_{\text{train}}^{\left(\text{NDS}\right)},\;P_{\text{test}}^{\left(\text{NDS}\right)}\cap P_{\text{train}}^{\left(\text{NDS}\right)}=\emptyset$$

#### 2.1.2 Known Drug Setting (KDS)

In this setting, test drugs already appear in the training set but only with a subset of their ADR associations. The task is to predict additional ADR links for these drugs. This scenario evaluates the model’s ability to identify underreported drug–ADR associations within a known entity space, supporting the completion and refinement of existing drug safety knowledge bases. Formally:


$$D_{\text{test}}^{\left(\text{KDS}\right)}=D_{\text{train}}^{\left(\text{KDS}\right)},\;R_{\text{test}}^{\left(\text{KDS}\right)}=R_{\text{train}}^{\left(\text{KDS}\right)},\;P_{\text{test}}^{\left(\text{KDS}\right)}\cap P_{\text{train}}^{\left(\text{KDS}\right)}=\emptyset$$

### 2.2 Dataset construction

#### 2.2.1 Data sources and preprocessing

We constructed our dataset from two public databases.

*ADR database*: ADReCS (Cai *et al.* 2015) is a comprehensive ADR ontology and knowledge base that integrates drug–ADR associations from multiple sources, including FAERS, SIDER4.1, MedDRA, and PubChem. We used the latest release (v3.3, June 2024).

*Drug database*: DrugBank (Knox *et al.* 2024) is a widely used curated drug database that provides diverse drug-related information. From DrugBank, we obtained drug identifiers, Simplified Molecular Input Line Entry System (SMILES) strings, and Anatomical Therapeutic Chemical (ATC) classification codes.

Drug–ADR associations were extracted from ADReCS and represented as a binary drug–ADR interaction matrix. ADReCS drug identifiers were mapped to DrugBank IDs using the official mapping table, after which SMILES strings and ATC codes were retrieved from DrugBank. ADRs were represented using their ADReCS identifiers obtained from the terminology table. Given that ADR datasets are inherently noisy (Alomar 2014, Maciejewski *et al.* 2017, Caballero and Quirce 2020), retaining ADRs with very few associated drugs would introduce spurious signals rather than true pharmacological associations. Therefore, referring to the data-cleaning strategy consistent with prior literature (Zhao *et al.* 2023), ADRs associated with fewer than 20 drugs were excluded. The final dataset contains 2088 drugs (hereafter referred to as ADR drugs) and 2062 ADRs.

#### 2.2.2 Dataset partitioning and construction

##### 2.2.2.1 NDS dataset

**Figure 2:**
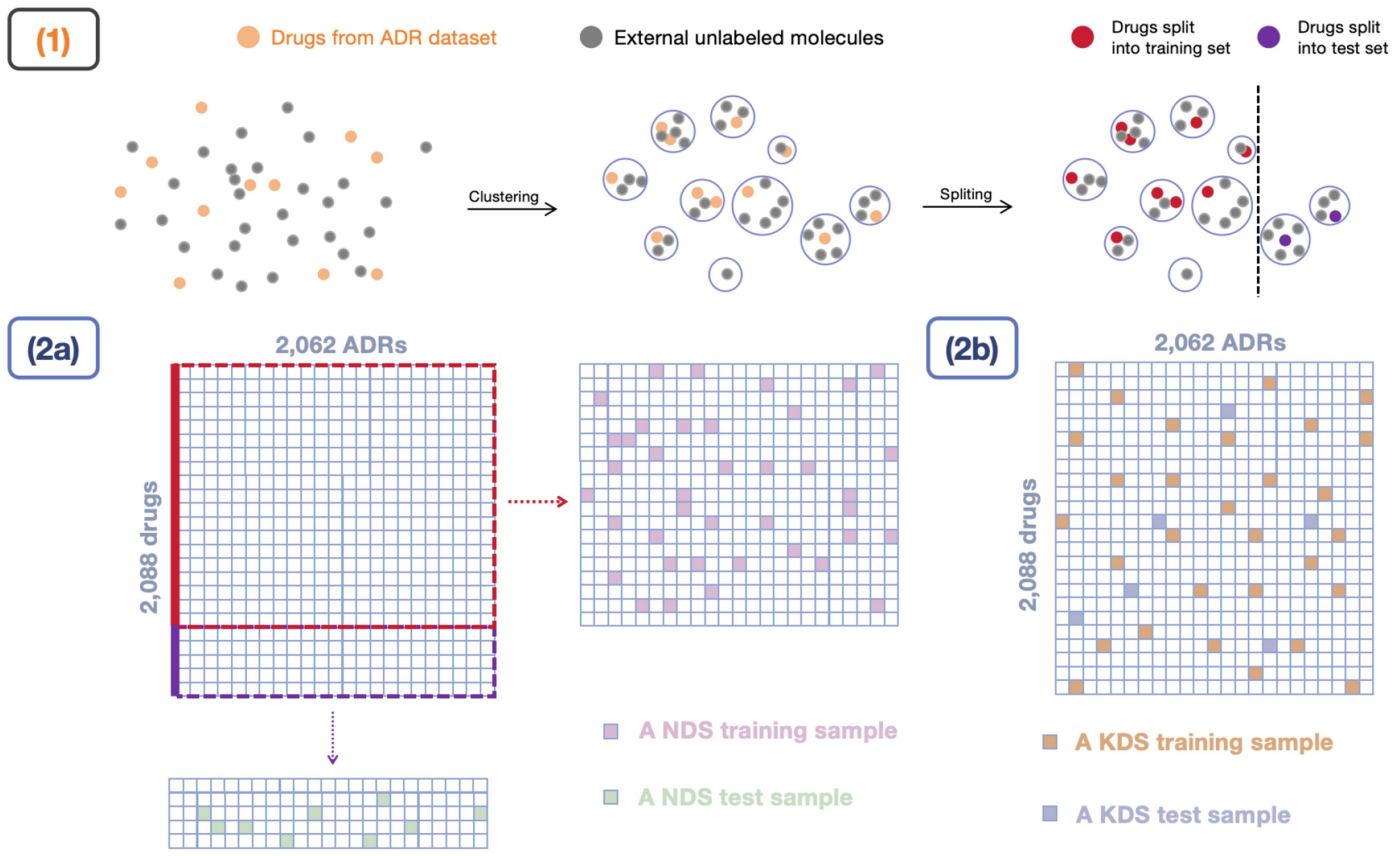
(1) Dataset partitioning. ADR drugs and external drugs are clustered into source and target domains, where ADR drugs in the source domain are used for training and those in the target domain for testing. (2) Dataset construction by forming drug-ADR pairs: (a) in NDS; (b) in KDS.

To better reflect real-world scenarios where novel drugs may follow a different distribution from training drugs, we introduce a distribution shift between training and test sets, inspired by Zeng *et al.* (2024). A schematic illustration is provided in Fig. 2(1). Specifically, 10 000 external drugs (marked in gray) from DrugBank—with no overlap with the 2088 labeled drugs and no recorded drug–ADR associations—were clustered together with the labeled drugs (orange) based on molecular similarity. The resulting clusters were split into source (80%) and target (20%) domains, yielding 1775 source-domain (red) and 313 target-domain (purple) labeled drugs for training and testing, respectively. Unlabeled target-domain drugs were additionally used for adversarial training. Details of the split strategy and domain shift characterization are provided in Supplementary Section 2, available as supplementary data at *Bioinformatics* online.

Training and test sets were constructed by pairing each drug with all ADRs (Fig. 2(2a)), yielding 1,775×2,062 and 313×2,062 candidate pairs (red and purple boxes), respectively. After downsampling negatives to balance classes, 232 821 training and 69 672 test pairs were obtained (pink and green cells). We adopted the 1:1 downsampling ratio following the protocols in prior ADR prediction studies (Zhang *et al.* 2021, He *et al.* 2025), while also mitigating the risk of mislabeling unobserved drug–ADR pairs that may represent unrecorded positives rather than true negatives.

##### 2.2.2.2 KDS dataset

As training and test sets share the same drugs, no drug-level partition is required. All drugs and ADRs are combined into candidate pairs (Fig. 2(2 b)), yielding 352 902 pairs after negative downsampling, randomly split into training (80%) and test (20%) sets (blue and orange cells).

### 2.3 Model architecture

**Figure 3:**
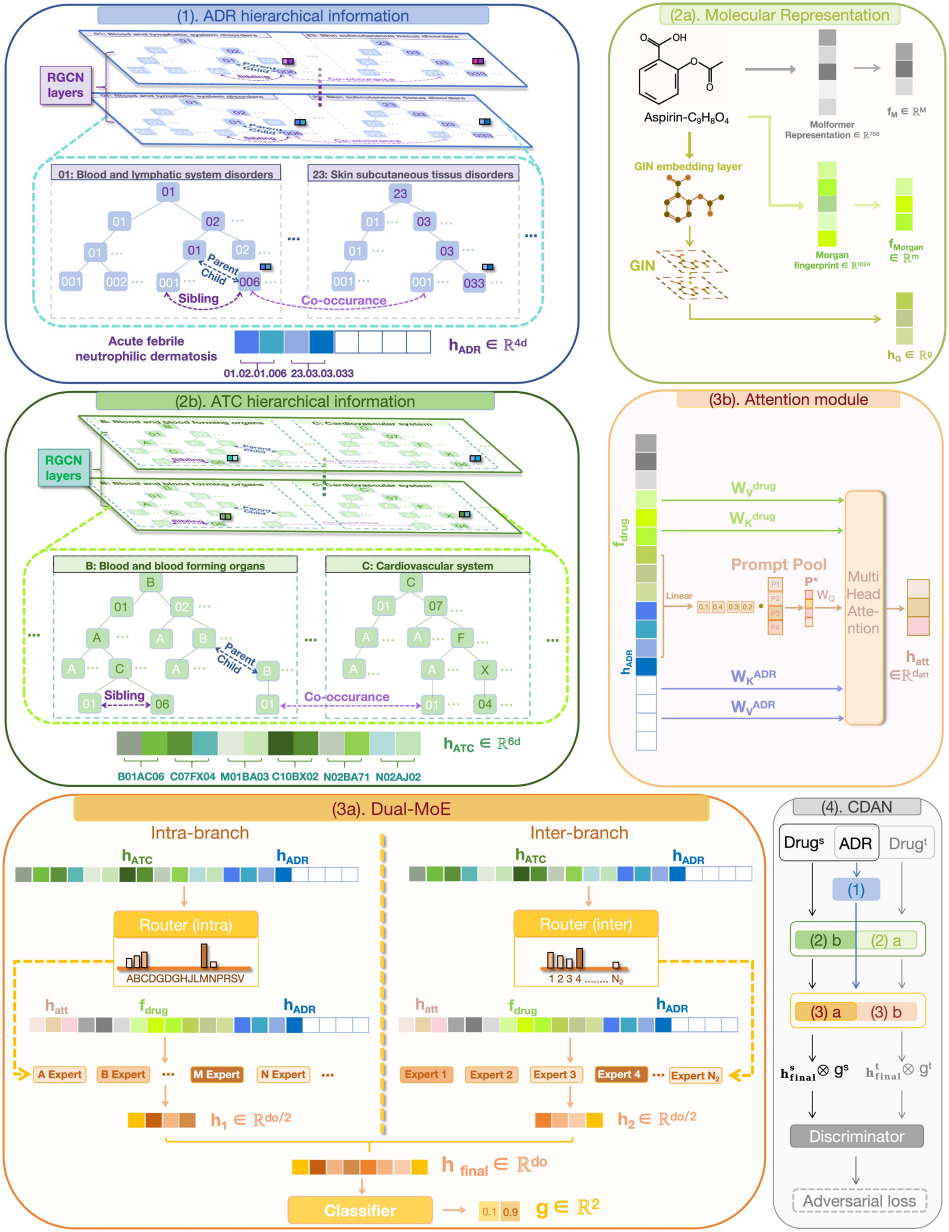
Overall framework of the proposed model in NDS. (1) ADR knowledge graph modeled using an R-GCN, exemplified by Acute febrile neutrophilic dermatosis. (2a) Drug molecular embedding fusing Morgan, GIN, and Molformer. (2b) ATC knowledge graph processed using an R-GCN, exemplified by Aspirin. (3a) Dual-MoE with ATC-guided intra-experts and general-purpose inter-experts. (3b) Attention module for the fusion of molecular and ADR representations. (4) CDAN module. In KDS, the Molformer representation and the CDAN module are not included.

Figure 3 presents an overview of the proposed framework, which consists of four main components:

- *ADR representation module*. An RGCN-based module that jointly exploits ADR hierarchical structure and co-occurrence relations through relation-aware message passing to learn informative ADR embeddings (Fig. 3(1)).
- *Drug representation module*. This module incorporates molecular representations (Fig. 3(2a)) to capture chemical properties and ATC-based representations (Fig. 3(2b)) to encode therapeutic context, which facilitates generalization to novel drugs.
- *Dual-MoE module*. A Dual Mixture-of-Experts architecture that models specialization within therapeutic categories while capturing global cross-category patterns (Fig. 3(3a, 3b)).
- *Domain adaptation module*. A CDAN-based module designed to mitigate distribution shifts between training drugs and novel drugs when predicting ADRs.

#### 2.3.1 ADR representation module

To capture hierarchical and empirical relationships among ADRs, we construct an ADR knowledge graph. In this graph, each ADR term is represented through its four-level ADReCS hierarchy, where nodes correspond to hierarchical categories. The initial node features are learnable embeddings, randomly initialized and jointly optimized with the R-GCN and subsequent modules during training. Three types of relations are incorporated in the ADR graph:

##### 2.3.1.1 Parent–child relations

Directed edges connect parent and child nodes in the hierarchy through *child-of* and reverse *parent-of* relations, enabling both upward aggregation of specific information and downward propagation of abstract knowledge.

##### 2.3.1.2 Sibling relations

Bidirectional *sibling-of* edges connect PT nodes under the same HLT. This design enhances semantic relatedness among bottom-level ADR entities within the same parent category.

##### 2.3.1.3 Co-occurrence relations

Beyond hierarchical relations, co-occurrence relations are incorporated by computing pairwise Jaccard similarity between PTs based on the sets of drugs linked to them. Based on empirical results, we retain the top 25% most similar node pairs as co-occurrence edges, using similarity scores as edge weights. Overly high thresholds produce dense graphs with noisy interactions, while overly low thresholds may remove informative co-occurrence relationships.

To model heterogeneous relations, we employ an R-GCN on a multi-relational graph G=(V,E), where each edge (i,j)∈E carries a relation type r∈R and scalar weight $w_{ij}\geq 0$. Let $N_{i}^{r}$ denote the neighbors of node *i* under relation *r*, and define the normalized edge weight ${\tilde{w}}_{ij}^{r}=w_{ij}/\sum_{k\in N_{i}^{r}}w_{ik}$, so that $\sum_{j\in N_{i}^{r}}{\tilde{w}}_{ij}^{r}=1$, ensuring the aggregated signal is scale-invariant with respect to node degree. The propagation rule of a single R-GCN layer is then


(1) $$h_{i}^{\left(l+1\right)}=\sigma \left(W_{\text{self}}^{\left(l\right)}h_{i}^{\left(l\right)}+\sum_{r\in R}\sum_{j\in N_{i}^{r}}{\tilde{w}}_{ij}^{r}W_{r}^{\left(l\right)}h_{j}^{\left(l\right)}\right),$$

Where $h_{i}^{\left(0\right)}$ is the initial node feature vector. The self-connection term $W_{\text{self}}^{\left(l\right)}h_{i}^{\left(l\right)}$ retains and refines the node’s own features, while the aggregation term applies a relation-specific transformation $W_{r}^{\left(l\right)}$ to capture the semantic role of each relation before accumulating neighbor representations. After stacking *L* layers, the final representation $h_{i}^{\left(L\right)}$ is passed to downstream modules.

The resulting embeddings of the lowest-level nodes are used to construct the ADR representation. Since an ADR may correspond to multiple ADReCS IDs, its representation $h_{\text{ADR}}$ is formed by concatenating the embeddings of the associated IDs (up to four, as this covers all ADRs in the dataset; see Supplementary Section 3, available as supplementary data at *Bioinformatics* online for details), with zero-padding applied when fewer IDs are available.

#### 2.3.2 Drug representation module

##### 2.3.2.1 Molecular representation

Each drug is represented using three complementary features: 1024-bit Morgan fingerprints (radius 2) for local substructures, GIN-based molecular graph representations for topological structure, and—in the NDS setting—Molformer features (Pan 2023) for general chemical representations learned from large-scale data to improve generalization to novel drugs. The final representation $f_{\text{drug}}\in R^{D}$ is obtained by concatenating these feature vectors.

##### 2.3.2.2 ATC representation

We construct an ATC knowledge graph following the same design as the ADR graph. Each drug is represented through its five-level ATC hierarchy, with parent–child relations defined along the hierarchy, sibling relations connecting level-5 codes sharing the same level-4 parent, and co-occurrence edges defined by pairwise Jaccard similarity between level-5 codes (top 25% most similar pairs retained as weighted edges, consistent with the ADR co-occurrence configuration). The graph is encoded via R-GCN to obtain node representations. A drug may have multiple ATC codes; its representation $h_{\text{ATC}}$ is formed by concatenating the embeddings of up to six ATC codes (zero-padded when necessary), as 97% of training drugs have six or fewer codes, balancing information retention against padding redundancy (see Supplementary Section 3, available as supplementary data at *Bioinformatics* online for details).

#### 2.3.3 Dual-MoE module

##### 2.3.3.1 Prompt cross-attention

To model the interaction between the drug representation and the ADR representation of a sample, we introduce a Prompt Cross-Attention module with a learnable prompt pool. For each drug–ADR pair, the drug and ADR representations are concatenated and fed into a selection network to generate weights over the prompt pool and produce an instance-specific prompt. The selected prompt serves as the query in a multi-head cross-attention mechanism, while the drug and ADR representations serve as the keys and values, yielding a representation $h_{\text{att}}$ that is used as input to the Dual-MoE module. We introduce this design because raw drug and ADR representations may contain modality-specific noise irrelevant to their interaction. The prompt pool constrains all interactions to be reconstructed within a structured space, acting as an information bottleneck that suppresses irrelevant features while retaining interaction-relevant signals.

##### 2.3.3.2 Dual-MoE

We propose a Dual-MoE architecture with two branches: an ATC-specific intra-expert branch capturing intra-class ADR patterns, and a globally shared inter-expert branch modeling cross-category dependencies. In both branches, routing networks take ATC and ADR representations as input, while experts receive the drug molecular representation, ADR representation, and attention module output.

*Intra-expert branch.* Each second-level ATC class is associated with a dedicated expert ($N_{1}=87$). For each drug, the routing network computes expert selection weights, which are then masked by the drug’s ATC category so that only the corresponding experts are activated. The masked weights are renormalized to aggregate selected expert outputs, producing $h_{1}\in R^{d_{o}/2}$.

*Inter-expert branch.* This branch captures dependencies shared across all drugs. A top-*k* sparse gating strategy activates only the highest-scoring experts per input, yielding sparse computation and reduced overhead.

To promote balanced utilization of the inter-experts and prevent routing collapse—where only a subset of experts receives the majority of inputs—we introduce a load-balancing loss:


(2) $$L_{\text{balance}}=N_{2}\cdot \sum_{i=1}^{N_{2}}f_{i}\cdot G_{i},$$

Where $N_{2}$ is the total number of inter-experts, and the product $f_{i}\cdot G_{i}$ jointly penalizes imbalance from two perspectives: the discrete activation frequency ($f_{i}$) and the continuous gating probability ($G_{i}$). Specifically, $f_{i}=\frac{1}{B}\sum_{t=1}^{B}1\left[\text{expert}\;i\in \text{top}-k\left(g_{\text{inter}}^{\left(t\right)}\right)\right]$ is the dispatch fraction of expert *i* over a batch of size *B*, where 1[·] returns 1 if expert *i* is among the *k* highest-scoring experts for sample *t* and 0 otherwise. Thus $f_{i}\in [0,1]$ measures the empirical activation rate of expert *i*; under perfect load balance $f_{i}=k/N_{2}$, and values substantially exceeding this indicate over-utilization. $G_{i}=\frac{1}{B}\sum_{t=1}^{B}g_{\text{inter},i}^{\left(t\right)}$ is the mean routing probability assigned to expert *i* across the batch, where $g_{\text{inter},i}^{\left(t\right)}$ denotes the gating score for expert *i* on sample *t*.

The outputs of the selected experts are aggregated using normalized routing weights, yielding the inter-branch representation $h_{2}\in R^{d_{o}/2}$. The intra- and inter-branch representations are concatenated to form the final embedding $h_{\text{final}}\in R^{d_{o}}$, which is passed to a classification head for binary ADR prediction.

#### 2.3.4 CDAN

To improve generalization to novel drugs under distribution shifts, we incorporate CDAN to learn domain-invariant representations.

Let $N_{s}$ labeled drug–ADR pairs in the training data form the source domain $D_{s}={\left\{(f_{\text{drug},i}^{s},{\text{ADR}}_{i},y_{i}^{s})\right\}}_{i=1}^{N_{s}}$, while $N_{t}$ drug–ADR pairs constructed from the 1,775 target-domain drugs and all ADRs constitute the target domain $D_{t}={\left\{(f_{\text{drug},j}^{t},{\text{ADR}}_{j})\right\}}_{j=1}^{N_{t}}$. A domain label d=0 is assigned to source samples and d=1 to target samples.

Samples from both domains are first processed by the feature encoder F(·) to obtain the representation $h_{\text{final}}$, which is then passed to the predictor G(·) to produce the class probability vector $\hat{y}$. Following CDAN, we construct a conditional representation $h_{\text{cond}}=h_{\text{final}}⊗\hat{y},$ which captures the interaction between feature representations and classifier predictions. The conditional representation is subsequently fed into a domain discriminator D(·) for domain classification.

The discriminator is trained to distinguish source and target samples, while the feature encoder is trained adversarially to confuse the discriminator. Through this adversarial process, the encoder learns representations that remain predictive for ADR classification while being invariant across domains, thereby improving generalization to the target domain.

### 2.4 Training objective

The ADR prediction loss is defined as the cross-entropy between the predicted and ground-truth ADR labels:


(3) $$L_{\text{main}}=E_{(f_{\text{drug}}^{s},\text{ADR},y)∼D_{s}}\left[-\sum_{c=1}^{2}y_{c}\;\text{log}({\hat{y}}_{c})\right].$$

The adversarial domain loss is defined as


(4) $$\begin{array}{cc} L_{\text{adv}}(F,G,D)=E_{(f_{\text{drug}}^{s},{\text{ADR}}^{s})∼D_{s}}\left[\text{log}\;D(h_{\text{cond}}^{s})\right] & \\ +E_{(f_{\text{drug}}^{t},{\text{ADR}}^{s})∼D_{t}}\left[\text{log}(1-D(h_{\text{cond}}^{t}))\right], & \end{array}$$

Where $h_{\text{cond}}^{s}$ and $h_{\text{cond}}^{t}$ denote the conditional representations of source and target samples, respectively.

The overall optimization for the NDS setting is formulated as a minimax objective between the feature extractor and predictor (F,G) and the domain discriminator *D*:


(5) $$\underset{F,G}{\min}\underset{D}{\max}\left(L_{\text{main}}+\lambda_{1}L_{\text{balance}}-\lambda_{2}L_{\text{adv}}\right),$$

Where $\lambda_{1}$ and $\lambda_{2}$ control the contributions of the load-balancing loss and the adversarial loss, respectively.

For the KDS setting, where domain adaptation is not applied, the training objective reduces to


(6) $$\underset{F,G}{\min}\left(L_{\text{main}}+\lambda_{1}L_{\;\text{balance}}\right).$$

## 3 Results and discussion

### 3.1 Overall performance

We compare against seven baselines spanning diverse methodological paradigms: a collaborative filtering approach (ML-CF) (Li *et al.* 2023), a method exploiting a different molecular representation strategy (Image-CNN) (Das and Mazumder 2025), a message passing network with gene expression features (BiMPADR) (Li *et al.* 2024a), a multi-modal heterogeneous GNN (PreciseADR) (Gao *et al.* 2024), and a large-scale biomedical knowledge graph method (KG-based) (Li *et al.* 2024b). We also include GCAP (Zhao *et al.* 2023) and OrganADR (Li *et al.* 2025), state-of-the-art models for closely related ADR-relevant tasks, namely ADR severity prediction and DDI-induced ADR prediction, respectively. We additionally include DrugBAN in NDS (Bai *et al.* 2023), a drug-target interaction prediction model leveraging CDAN. Task-specific adaptations, environment and training protocol and hyperparameter configurations are provided in the Supplementary Section 4, available as supplementary data at *Bioinformatics* online. Hyperparameter Settings of the proposed model are provided in the Supplementary Section 14, available as supplementary data at *Bioinformatics* online.

Results are presented in Tables 1 and 2. Our model consistently achieves the best performance across nearly all metrics, improving F1 by 4.7% (NDS) and 3.7% (KDS) over the strongest baseline, with lower variance across runs indicating better reproducibility. Notably, it achieves higher recall than all baselines while maintaining near-top precision, a property particularly desirable for drug safety monitoring where missing true ADRs is critical. Statistical analysis confirms that the majority of *P*value fall below 0.05 (see Supplementary Section 5, available as supplementary data at *Bioinformatics* online for details). Computational cost is discussed in Supplementary Section 6, available as supplementary data at *Bioinformatics* online; in brief, our model incurs moderately longer training time as a trade-off for improved performance, while inference poses no practical barrier to deployment.

**Table 1:** Performance comparison with baselines in NDS (mean  ±  std).

Model	F1	ROC-AUC	PR-AUC	Precision	Recall	Accuracy
GCAP	0.6952 ± 0.0099	0.8121 ± 0.0063	0.8121 ± 0.0056	0.7901 ± 0.0085	0.6216 ± 0.0200	0.7279 ± 0.0076
OrganADR	0.6851 ± 0.0139	0.7719 ± 0.0071	0.7603 ± 0.0085	0.6479 ± 0.0089	0.7214 ± 0.0228	0.6966 ± 0.0070
PreciseADR	0.7179 ± 0.0092	0.7791 ± 0.0054	0.7742 ± 0.0043	0.7478 ± 0.0114	0.6913 ± 0.0148	0.7287 ± 0.0084
ML-CF	0.6550 ± 0.0115	0.7957 ± 0.0041	0.7959 ± 0.0051	0.7915 ± 0.0095	0.5597 ± 0.0144	0.7057 ± 0.0077
Image-CNN	0.6625 ± 0.0159	0.7957 ± 0.0102	0.8020 ± 0.0114	0.8049 ± 0.0243	0.5640 ± 0.0248	0.7133 ± 0.0130
BiMPADR	0.7157 ± 0.0086	0.7924 ± 0.0054	0.7907 ± 0.0045	0.7501 ± 0.0113	0.6852 ± 0.0224	0.7282 ± 0.0071
DrugBAN	0.7071 ± 0.0070	0.7823 ± 0.0045	0.7798 ± 0.0056	0.7234 ± 0.0107	0.6924 ± 0.0090	0.7156 ± 0.0066
KG-based	0.7282 ± 0.0111	0.7904 ± 0.0067	0.7855 ± 0.0067	0.6893 ± 0.0142	0.7780 ± 0.0187	0.7189 ± 0.0086
Ours	0.7753 ± 0.0063	0.8483 ± 0.0021	0.8498 ± 0.0026	0.7707 ± 0.0096	0.7803 ± 0.0090	0.7753 ± 0.0056

Bold values indicate the best performance. Standard deviations are computed over five runs with different seeds.

**Table 2:** Performance comparison with baselines in KDS (mean  ±  std).

Model	F1	ROC-AUC	PR-AUC	Precision	Recall	Accuracy
GCAP	0.8127 ± 0.0012	0.9008 ± 0.0009	0.8823 ± 0.0013	0.8003 ± 0.0089	0.8269 ± 0.0076	0.8219 ± 0.0023
OrganADR	0.7815 ± 0.0032	0.8719 ± 0.0042	0.8530 ± 0.0033	0.7653 ± 0.0103	0.8010 ± 0.0087	0.7847 ± 0.0052
PreciseADR	0.8178 ± 0.0013	0.9043 ± 0.0020	0.8802 ± 0.0018	0.8051 ± 0.0045	0.8306 ± 0.0062	0.8268 ± 0.0020
ML-CF	0.8132 ± 0.0021	0.8997 ± 0.0019	0.8794 ± 0.0018	0.7958 ± 0.0043	0.8224 ± 0.0057	0.8210 ± 0.0029
Image-CNN	0.6494 ± 0.0034	0.7921 ± 0.0044	0.7804 ± 0.0048	0.7796 ± 0.0167	0.5655 ± 0.0121	0.7194 ± 0.0023
BiMPADR	0.8155 ± 0.0021	0.9079 ± 0.0016	0.8896 ± 0.0011	0.8173 ± 0.0049	0.8140 ± 0.0058	0.8277 ± 0.0034
KG-based	0.8005 ± 0.0013	0.8873 ± 0.0020	0.8642 ± 0.0021	0.7842 ± 0.0032	0.8174 ± 0.0043	0.8157 ± 0.0034
Ours	0.8555 ± 0.0005	0.9330 ± 0.0003	0.9172 ± 0.0005	0.8171 ± 0.0023	0.8971 ± 0.0031	0.8581 ± 0.0004

Bold values indicate the best performance. Standard deviations are computed over five runs with different seeds.

### 3.2 Ablation study

**Figure 4:**
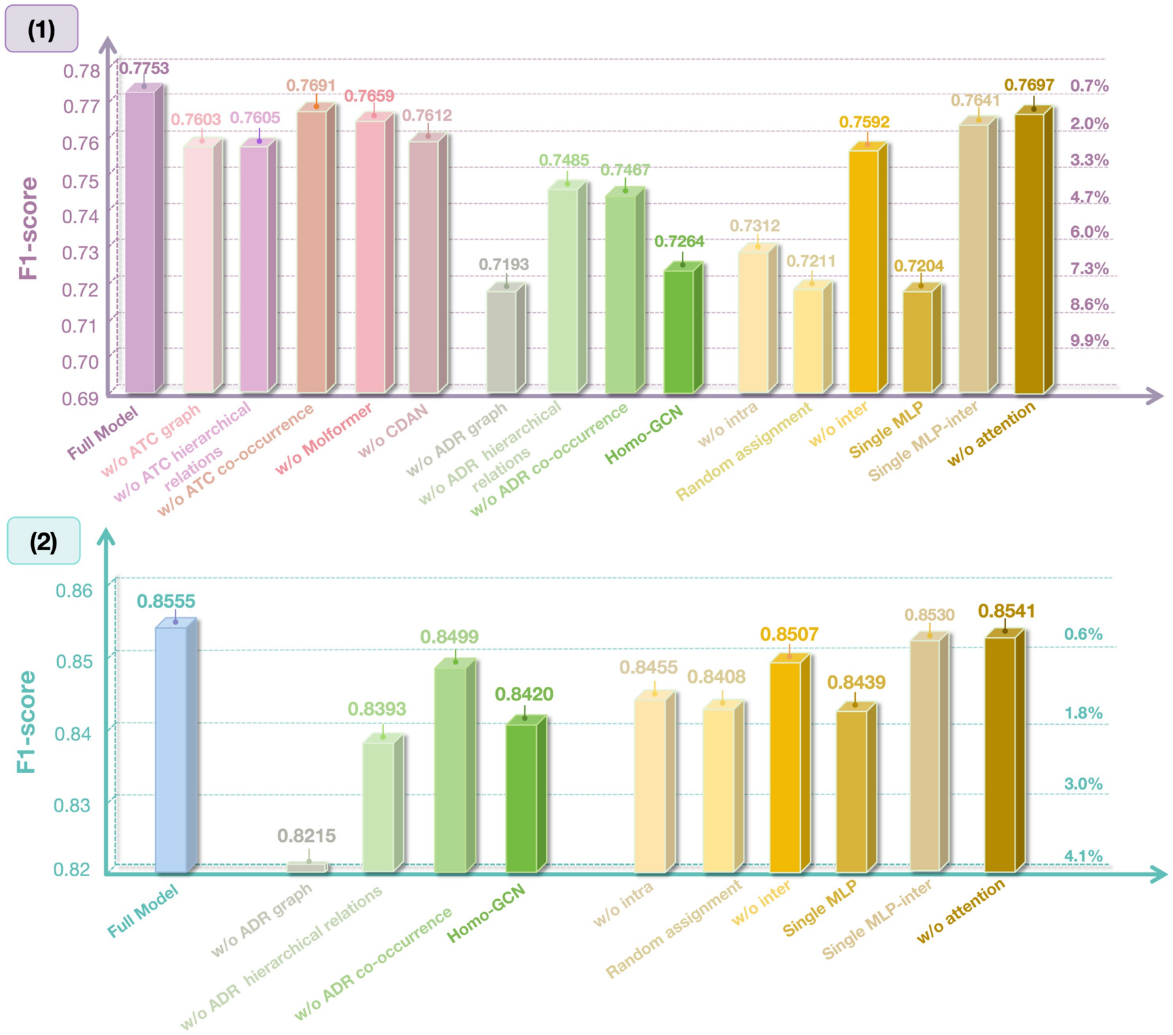
Ablation results (F1 score) for the proposed model: (1) in NDS; (2) in KDS. The percentages annotated above each dashed line indicate the relative performance drop compared with the full model.

To evaluate each component, we conduct ablation studies under both settings and report F1 results in Fig. 4 (see Table S11 and S12, available as supplementary data at *Bioinformatics* online for details). For ATC representations, variants include removing the ATC graph entirely (w/o ATC graph), removing hierarchical relations (w/o ATC hierarchical relations), and removing co-occurrence edges (w/o ATC co-occurrence). The same three variants are applied to ADR representations, with an additional comparison replacing R-GCN with a homogeneous GCN (Homo-GCN) to assess relation-specific message passing. For the Dual-MoE module, we remove the intra-expert branch (w/o intra) and replace ATC-guided routing with random assignment (Random assignment) to isolate the contribution of structured routing; we also substitute the entire branch with a parameter-matched single MLP (Single MLP) for a macroscopic comparison of overall expert specialization effectiveness. The same MLP substitution and full removal are applied to the inter-expert branch (Single MLP-inter; w/o inter). Finally, we ablate the prompt-based attention module (w/o attention), Molformer (w/o Molformer), and CDAN (w/o CDAN).

#### 3.2.1 ATC graph module

Removing the ATC graph reduces F1 by 1.5% in NDS, indicating that higher-level ATC categories help establish pharmacological links between novel and known drugs. Removing ATC hierarchical relations causes a comparable decline, consistent with novel drugs primarily relying on hierarchical links to connect to the existing ATC structure. Removing co-occurrence edges also degrades performance, as these edges enable novel drugs to capture cross-category ADR similarities through shared neighbors, while hierarchical relations mainly connect drugs within the same top-level category.

#### 3.2.2 ADR representation module

Removing the ADR graph causes the largest F1 drop in both settings (5.6% in NDS, 3.4% in KDS), making it the most critical component. This demonstrates the importance of structured, relation-aware ADR representations for capturing semantic similarity and empirical co-occurrence among adverse reactions. Replacing R-GCN with Homo-GCN also leads to substantial degradation, confirming the necessity of relation-specific message passing. Hierarchical relations are less influential than co-occurrence relations in NDS but more influential in KDS, because hierarchical relations are sparse and semantically strict, requiring reliable drug embeddings—harder to satisfy for novel drugs in NDS. Co-occurrence relations, being denser, allow signals to propagate through indirect connections even with imprecise drug embeddings, making them more robust under NDS.

#### 3.2.3 Dual-MoE module

Replacing Dual-MoE with a parameter-matched single MLP causes a large performance drop, showing that gains arise from expert specialization rather than parameter count, further supported by the analogous ablation on the inter-expert branch. Removing either branch leads to consistent drops in both settings, with the intra-expert branch playing a more prominent role. Replacing ATC-guided routing with random assignment also causes a large drop, suggesting that improvement stems from pharmacologically meaningful expert assignment rather than hard specialization alone.

#### 3.2.4 Auxiliary modules

Removing CDAN causes a 1.5% F1 drop in NDS, where distribution shifts are more severe by design. Molformer contributes 0.9% by improving chemical representation and generalization to novel drugs. The prompt-based attention module provides smaller but consistent gains in both settings, indicating its role in suppressing interaction-irrelevant noise.

Additionally, the ablation results show larger performance drops for all the components in NDS than in KDS. This is likely because the model has no prior exposure to novel drugs in NDS during training, making their representations less reliable and causing the model to rely more heavily on each component to extract and propagate useful signals. Interestingly, we observe a slight divergence between F1-score and AUC when CDAN is removed in the NDS ablation study, with further discussion provided in Supplementary Section 7.1, available as supplementary data at *Bioinformatics* online.

### 3.3 Impact of ADR frequency buckets

Detecting infrequent ADRs is critical in pharmacovigilance due to their underreporting and potential severity. To evaluate performance across ADR rarity, we stratified ADRs into five frequency buckets based on incidence rate: Very Common (≥10%), Common-High (5–10%), Common-Medium (2–5%), Common-Low (1–2%), and Uncommon (0.1–1%). The three baselines with the highest overall F1 scores were included for comparison.

**Figure 5:**
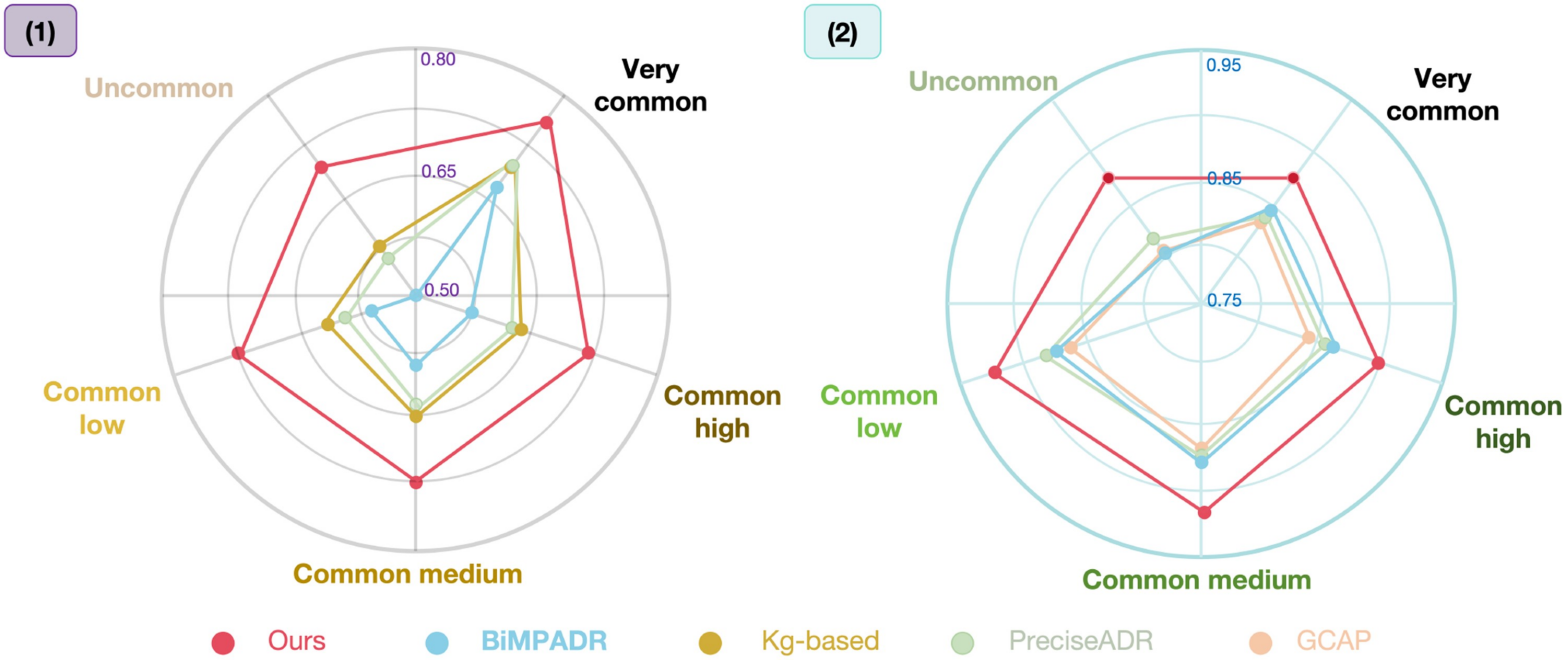
Comparison of ROC-AUC of different ADR rarity buckets with baselines: (1) in NDS; (2) in KDS.

As shown in Figure 5 (see Table S13, available as supplementary data at *Bioinformatics* online for details), our method consistently outperforms all baselines across all rarity groups under both NDS and KDS settings, with the advantage widening for low-frequency and uncommon ADRs. This is attributable to the hierarchical structure of the ADR graph: in a flat graph, rare ADR nodes with few associated drugs are easily overshadowed by high-degree nodes during message passing, whereas anchoring rare ADRs within the ADReCS taxonomy allows the R-GCN to inherit semantic context from parent and sibling nodes, compensating for data scarcity and producing more discriminative representations.

We set a threshold of ≥20 drugs to ensure label reliability and evaluation stability. To further assess the sensitivity of this choice, we conducted an additional experiment using ≥10 drugs, designated the additionally included ADRs as the *rare* bucket (Supplementary Section 8.2, available as supplementary data at *Bioinformatics* online). Under KDS, consistent conclusions are drawn; under NDS, however, performance gains diminish as all methods approach their practical limits, likely due to the compounded difficulty of generalizing to novel drugs and ADRs with minimal training signal, as well as potential label noise in pharmacovigilance databases—both left for future work.

### 3.4 Performance validation on SIDER

As introduced in Section 2.2, ADReCS integrates drug-ADR associations from multiple sources (FAERS, SIDER, DailyMed) and serves as the primary dataset throughout this study. However, the integration process may introduce differences relative to the original databases due to additional extraction and cleaning procedures. To verify that the reported improvements are attributable to the proposed method rather than dataset-specific processing choices, we conduct additional experiments directly on the SIDER (Kuhn *et al.* 2016) dataset. Data sources and preprocessing are provided in Supplementary Section 9, available as supplementary data at *Bioinformatics* online.

Results are presented in Tables 3 and 4. Our model again outperforms all baselines, achieving F1 improvements of 4.4% under NDS and 3.6% under KDS. The slightly smaller gains compared to ADReCS (4.7% and 3.7%) are likely due to SIDER’s more limited drug and ADR variety, which reduces the margin for improvement; the larger advantages on the more complex ADReCS dataset further confirm our model’s strength in challenging settings. Consistent precision–recall balance is maintained across both datasets, and the improvements observed confirm that the reported gains stem from the proposed method rather than dataset-specific processing choices.

**Table 3:** Performance comparison with baselines on SIDER in NDS (mean  ±  std).

Model	F1	ROC-AUC	PR-AUC	Precision	Recall	Accuracy
GCAP	0.7008 ± 0.0069	0.8179 ± 0.0043	0.8151 ± 0.0056	0.8191 ± 0.0099	0.6046 ± 0.0160	0.7321 ± 0.0045
OrganADR	0.6892 ± 0.0099	0.7754 ± 0.0067	0.7638 ± 0.0069	0.6839 ± 0.0123	0.6974 ± 0.0141	0.6996 ± 0.0071
PreciseADR	0.7240 ± 0.0082	0.7833 ± 0.0039	0.7791 ± 0.0064	0.7546 ± 0.0109	0.7013 ± 0.0132	0.7317 ± 0.0072
ML-CF	0.6620 ± 0.0059	0.7967 ± 0.0028	0.7988 ± 0.0055	0.7993 ± 0.0096	0.5665 ± 0.0154	0.7169 ± 0.0052
Image-CNN	0.6709 ± 0.0090	0.8067 ± 0.0075	0.8102 ± 0.0013	0.8039 ± 0.0198	0.5800 ± 0.0203	0.7196 ± 0.0099
BiMPADR	0.7189 ± 0.0056	0.7944 ± 0.0043	0.7967 ± 0.0069	0.7441 ± 0.0101	0.6992 ± 0.0149	0.7282 ± 0.0061
KG-based	0.7362 ± 0.0063	0.7929 ± 0.0067	0.7940 ± 0.0079	0.7209 ± 0.0099	0.7476 ± 0.0137	0.7255 ± 0.0068
Ours	0.7800 ± 0.0067	0.8483 ± 0.0033	0.8498 ± 0.0049	0.7707 ± 0.0096	0.7803 ± 0.0157	0.7753 ± 0.0066

Bold values indicate the best performance. Standard deviations are computed over five runs with different seeds.

**Table 4:** Performance comparison with baselines on SIDER in KDS (mean  ±  std).

Model	F1	ROC-AUC	PR-AUC	Precision	Recall	Accuracy
GCAP	0.8097 ± 0.0023	0.8996 ± 0.0003	0.8901 ± 0.0003	0.7963 ± 0.0103	0.8147 ± 0.0176	0.8156 ± 0.0013
OrganADR	0.7855 ± 0.0020	0.8734 ± 0.0033	0.8570 ± 0.0031	0.7783 ± 0.0183	0.7941 ± 0.0271	0.7868 ± 0.0042
PreciseADR	0.8169 ± 0.0017	0.9048 ± 0.0004	0.8939 ± 0.0002	0.8081 ± 0.0095	0.8286 ± 0.0122	0.8229 ± 0.0019
ML-CF	0.8078 ± 0.0020	0.8962 ± 0.0013	0.8753 ± 0.0026	0.7734 ± 0.0102	0.8362 ± 0.0227	0.8188 ± 0.0039
Image-CNN	0.6464 ± 0.0033	0.7910 ± 0.0024	0.7801 ± 0.0039	0.7527 ± 0.0132	0.5835 ± 0.0208	0.7163 ± 0.0033
BiMPADR	0.8132 ± 0.0011	0.9039 ± 0.0008	0.8846 ± 0.0013	0.8037 ± 0.0089	0.8201 ± 0.0138	0.8253 ± 0.0020
KG-based	0.7965 ± 0.0019	0.8803 ± 0.0009	0.8672 ± 0.0012	0.7703 ± 0.0099	0.8225 ± 0.0142	0.8136 ± 0.0021
Ours	0.8529 ± 0.0010	0.9298 ± 0.0001	0.9298 ± 0.0001	0.8511 ± 0.0095	0.8557 ± 0.0132	0.8522 ± 0.0016

Bold values indicate the best performance. Standard deviations are computed over five runs with different seeds.

### 3.5 Robustness analysis under data sparsity

**Figure 6:**
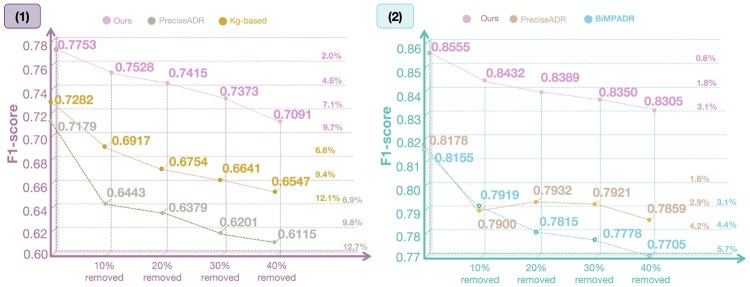
F1 performance of our model and the two strongest baselines under varying degrees of training data sparsity: (1) in NDS; (2) in KDS. The percentages annotated above the dashed lines indicate the relative performance drop compared with the full dataset.

To evaluate robustness under data sparsity, we simulate training-set reductions by removing associations of 10–40% of randomly selected drugs (NDS) or randomly removing 10%–40% of drug–ADR pairs (KDS), training our model alongside the two strongest baselines. As shown in Fig. 6 (see Table S15 and S16, available as supplementary data at *Bioinformatics* online for details), our model consistently outperforms the baselines at all sparsity levels with slower performance degradation, attributable to the ADR and ATC relational networks transforming isolated entities into an interconnected topological structure that leverages structural priors to compensate for missing data.

### 3.6 Evaluation under class imbalance

To evaluate robustness under class imbalance, we test on progressively skewed test sets (training remains 1:1) by increasing the negative-to-positive ratio from 1:1 to 19:1 (NDS) and 24:1 (KDS), reporting Hit@5, MRR, and balanced accuracy.

**Figure 7:**
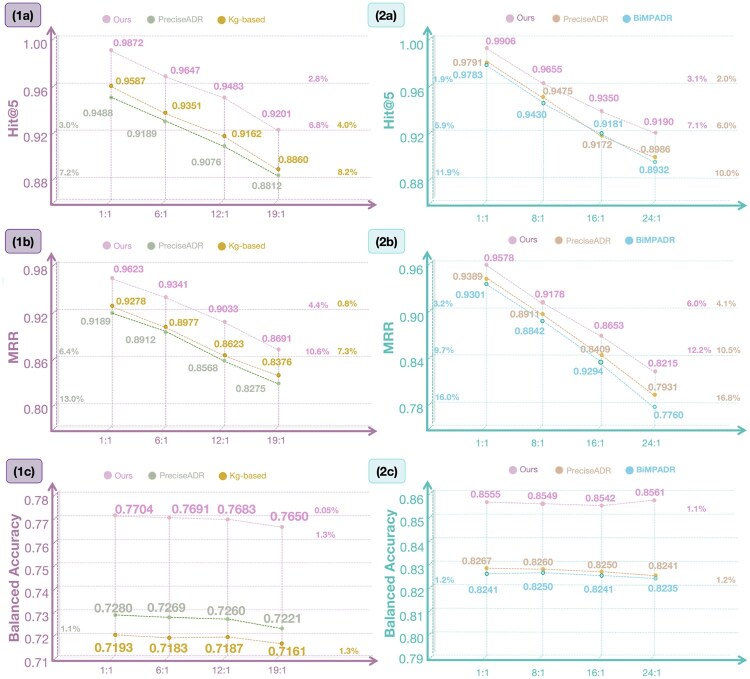
Performance of the proposed model and the two strongest baselines under progressively increasing class imbalance ratios: (1) in NDS; (2) in KDS. Panels (a), (b), and (c) report Hit@5, MRR, and Balanced Accuracy, respectively. The percentages annotated above the dashed lines indicate the relative performance drop compared with 1:1 ratio.

Results are shown in Fig. 7. Hit@5 and MRR decrease moderately with increasing imbalance due to larger test set difficulty, yet remain strong even at the most challenging ratios (Hit@5 ≥ 0.92; MRR: 0.87 on NDS, 0.82 on KDS). Balanced accuracy stays relatively stable across all ratios, indicating that the model preserves discriminative capability without degenerating into a majority-class predictor. Our method consistently outperforms the strongest baselines under all imbalance settings, confirming robustness in realistic skewed-distribution scenarios.

### 3.7 Interpretability and visualization

**Figure 8:**
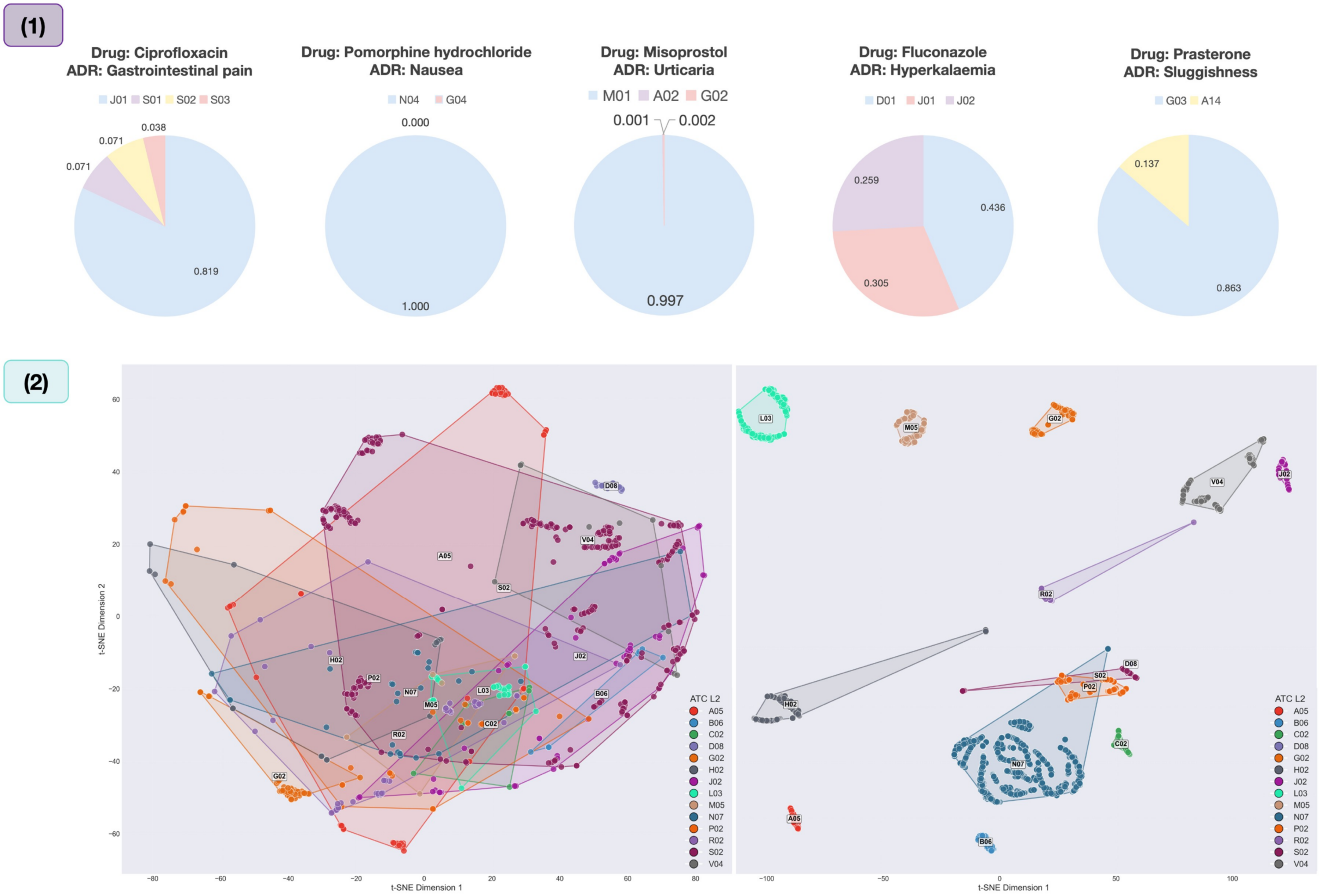
Interpretability and visualization results in NDS. (1) Routing weight analysis of the intra-expert router on five positive drug-ADR pairs. (2) t-SNE visualizations of the learned feature spaces for intra-experts (upper) and inter-experts (lower).

To evaluate whether the intra-expert router can identify the most relevant therapeutic or pharmacological subgroup for a given ADR when a drug belongs to multiple second-level ATC categories, we analyzed its routing weight distribution. We randomly selected five positive pairs in NDS for detailed analysis, with routing weight distributions shown in Fig. 8(a), and discuss one representative case here (see Supplementary Section 11, available as supplementary data at *Bioinformatics* online for additional cases). Ciprofloxacin (Knox *et al.* 2024) belongs to systemic class J01 and topical classes S01, S02, and S03. For gastrointestinal pain, the router assigns a high weight to J (0.8192), consistent with evidence that systemic ciprofloxacin commonly causes gastrointestinal ADRs, while ophthalmic or otic formulations mainly induce localized reactions (Ball 1986, U.S. Food and Drug Administration 2017, Limited 2018). Overall, the router provides an interpretable gating mechanism by assigning differential weights that reflect the most likely ADR source and guide expert selection.

In the MoE architecture, each sample yields two representations: intra-expert representations and inter-expert representations. To examine whether intra-experts capture specialized patterns while inter-experts encode more distributed information, we visualize both using t-SNE (Fig. 8b). For each ATC L1 category, one L2 subcategory was randomly selected from the test set to avoid overplotting while ensuring full L1 coverage. The upper and lower panels depict intra- and inter-expert representations, respectively, for the same samples, with each ATC L2 category distinctly colored and bounded by a convex hull.

The sharp spatial separation in the intra-expert space captures class-specific toxicological signatures, reflecting how distinct drug lineages systematically drive their characteristic ADR profiles. Conversely, the intertwined structure in the inter-expert space reveals shared mechanistic underpinnings across pharmacological categories. This dual structure confers simultaneous class-discriminative and globally integrative representation, enabling accurate prediction of class-characteristic ADRs while facilitating cross-class knowledge transfer to identify toxicity patterns inaccessible to a purely class-siloed framework.

Beyond the above analyses, we present three case studies in Supplementary Section 11, available as supplementary data at *Bioinformatics* online, using the strongest baseline under NDS as the reference for comparison to provide instance-level interpretable evidence for the mechanisms underlying the model’s predictive improvements.

### 3.8 Impact of hierarchical granularity

We further investigate how the granularity of hierarchical information affects model performance by comparing ATC embeddings at the fifth-, fourth-, and third-level, and ADR embeddings at the fourth-, third-, and second-level. Results show that coarser hierarchy levels lead to decreased performance, indicating that finer-grained representations are beneficial for predictions (see Supplementary Section 12, available as supplementary data at *Bioinformatics* online for details).

### 3.9 Parameter sensitivity and robustness analysis

To evaluate the robustness of our model to hyperparameter variations, we perform sensitivity analyses on the number of MoE inter-experts (activations equal to half the experts), the top-*k* activation, the load-balancing loss weight $\lambda_{1}$, the CDAN loss weight $\lambda_{2}$, the ADR Jaccard co-occurrence threshold, and the ATC Jaccard co-occurrence threshold with the results presented in Supplementary Section 13, available as supplementary data at *Bioinformatics* online Figure 1 and 2. The results show minor fluctuations and demonstrate that the model remains robust to these hyperparameter changes.

## 4 Conclusion

In this work, we propose a computational framework for ADR prediction that mitigates the challenges of ADR representation and novel-drug generalization. By coupling predefined ADR hierarchical structures with co-occurrence patterns and modeling with RGCN, our module encodes shared ADR semantic properties in the structural prior while also capturing empirical dependencies, resulting in meaningful and informative ADR representations. Incorporating hierarchical ATC information allows us to build meaningful connections and facilitate knowledge transfer between novel and existing drugs, and CDAN also helps to address out-of-distribution challenges for novel drugs. The introduction of a Dual-MoE architecture further enables the model to simultaneously capture specialized patterns and global dependencies. Extensive evaluations demonstrate that our approach consistently outperforms state-of-the-art baselines across different settings with superior precision-recall balance, exhibits improved detection performance for uncommon ADRs, while maintaining exceptional stability under data sparsity and providing meaningful interpretations that underscore its practical utility.

## Supplementary Material

btag494_Supplementary_Data

## Data Availability

The datasets supporting this study are publicly available: ADReCS (version 3.3, June 2024), available at https://bioinf.xmu.edu.cn/ADReCS and DrugBank at https://go.drugbank.com/.
