# Supplementary material for: Informative relational learning for adverse reaction prediction with enhanced generalization to novel drugs
Source: Bioinformatics. 2026 Jul 2;42(7):btag494. doi: 10.1093/bioinformatics/btag494 (PMC13364676; doi:10.1093/bioinformatics/btag494)
Supplement: btag494_Supplementary_Data [file btag494_supplementary_data.zip › 10-Jul-2026_061443_Supplementary_information.pdf]

Supplementary Information

Informative Relational Learning for Adverse Reaction Prediction with Enhanced Generalization to Novel Drugs

Shuge Sun[1], Dalin Zhang[1,*], Hongjun Chu[1], and Xinyi Gong[1]

[1]Space Information Research Institute, Hangzhou Dianzi University, Baiyang, Hangzhou, 310018, Zhejiang, China

***Corresponding author:** zhangdalin@hdu.edu.cn

1 Supplementary Tables for ADR and ATC Hierarchies

Table 1: **Complete list of the 26 first-level SOCs in the ADReCS, with two-digit codes and descriptions.**

Code	SOC Description
01	Blood and lymphatic system disorders
02	Cardiac disorders
03	Congenital, familial and genetic disorders
04	Ear and labyrinth disorders
05	Endocrine disorders
06	Eye disorders
07	Gastrointestinal disorders
08	General disorders and administration site conditions
09	Hepatobiliary disorders
10	Immune system disorders
11	Infections and infestations
12	Injury, poisoning and procedural complications
13	Investigations
14	Metabolism and nutrition disorders
15	Musculoskeletal and connective tissue disorders
16	Neoplasms benign, malignant and unspecified (incl cysts and polyps)
17	Nervous system disorders
18	Pregnancy, puerperium and perinatal conditions

Continued on next page

Table 1 continued from previous page

Code	System Organ Class (SOC) Description
19	Psychiatric disorders
20	Renal and urinary disorders
21	Reproductive system and breast disorders
22	Respiratory, thoracic and mediastinal disorders
23	Skin and subcutaneous tissue disorders
24	Vascular disorders
25	Surgical and medical procedures
26	Social circumstances

Table 2: **ATC Classification System: Hierarchical Overview.** First-level anatomical main groups with all corresponding second-level therapeutic/pharmacological subgroups.

First Level (Code and Description)	Second Levels (Code and Description)
A Alimentary tract and metabolism	A01 Stomatological preparations
	A02 Drugs for acid related disorders
	A03 Drugs for functional gastrointestinal disorders
	A04 Antiemetics and antinauseants
	A05 Bile and liver therapy
	A06 Drugs for constipation
	A07 Antidiarrheals, intestinal antiinflammatory/antiinfective agents
	A08 Antiobesity preparations, excl. diet products
	A09 Digestives, incl. enzymes
	A10 Drugs used in diabetes
	A11 Vitamins
	A12 Mineral supplements
	A13 Tonics
	A14 Anabolic agents for systemic use
	A15 Appetite stimulants
	A16 Other alimentary tract and metabolism products
B Blood and blood forming organs	B01 Antithrombotic agents
	B02 Antihemorrhagics
	B03 Antianemic preparations
	B05 Blood substitutes and perfusion solutions
	B06 Other hematological agents
C Cardiovascular system	C01 Cardiac therapy
	C02 Antihypertensives
	C03 Diuretics
	C04 Peripheral vasodilators
	C05 Vasoprotectives
	C07 Beta blocking agents

Continued on next page

Table 2 continued from previous page

First Level (Code and Description)	Second Levels (Code and Description)
	C08 Calcium channel blockers<br>C09 Agents acting on the renin-angiotensin system<br>C10 Lipid modifying agents
D Dermatologicals	D01 Antifungals for dermatological use<br>D02 Emollients and protectives<br>D03 Preparations for treatment of wounds and ulcers<br>D04 Antipruritics, incl. antihistamines, anesthetics, etc.<br>D05 Antipsoriatics<br>D06 Antibiotics and chemotherapeutics for dermatological use<br>D07 Corticosteroids, dermatological preparations<br>D08 Antiseptics and disinfectants<br>D09 Medicated dressings<br>D10 Anti-acne preparations<br>D11 Other dermatological preparations
G Genito-urinary system and sex hormones	G01 Gynecological antiinfectives and antiseptics<br>G02 Other gynecologicals<br>G03 Sex hormones and modulators of the genital system<br>G04 Urologicals
H Systemic hormonal preparations, excl. sex hormones and insulins	H01 Pituitary and hypothalamic hormones and analogues<br>H02 Corticosteroids for systemic use<br>H03 Thyroid therapy<br>H04 Pancreatic hormones<br>H05 Calcium homeostasis
J Antiinfectives for systemic use	J01 Antibacterials for systemic use<br>J02 Antimycotics for systemic use<br>J04 Antimycobacterials<br>J05 Antivirals for systemic use<br>J06 Immune sera and immunoglobulins<br>J07 Vaccines
L Antineoplastic and immunomodulating agents	L01 Antineoplastic agents<br>L02 Endocrine therapy<br>L03 Immunostimulants<br>L04 Immunosuppressants
M Musculo-skeletal system	M01 Antiinflammatory and antirheumatic products<br>M02 Topical products for joint and muscular pain<br>M03 Muscle relaxants<br>M04 Antigout preparations<br>M05 Drugs for treatment of bone diseases

Continued on next page

Table 2 continued from previous page

First Level (Code and Description)	Second Levels (Code and Description)
	M09 Other drugs for disorders of the musculo-skeletal system
N Nervous system	N01 Anesthetics
	N02 Analgesics
	N03 Antiepileptics
	N04 Anti-parkinson drugs
	N05 Psycholeptics
	N06 Psychoanaleptics
	N07 Other nervous system drugs
P Antiparasitic products, insecticides and repellents	P01 Antiprotozoals
	P02 Anthelmintics
	P03 Ectoparasiticides, incl. scabicides, insecticides and repellents
R Respiratory system	R01 Nasal preparations
	R02 Throat preparations
	R03 Drugs for obstructive airway diseases
	R05 Cough and cold preparations
	R06 Antiallergics, excl. systemic corticosteroids
	R07 Other respiratory system products
S Sensory organs	S01 Ophthalmologicals
	S02 Otologicals
	S03 Ophthalmological and otological preparations
V Various	V01 Allergens
	V03 All other therapeutic products
	V04 Diagnostic agents
	V06 General nutrients
	V07 All other non-therapeutic products
	V08 Contrast media
	V09 Diagnostic radiopharmaceuticals
	V10 Therapeutic radiopharmaceuticals
	V20 Surgical dressings

Table 3: **ATC classification hierarchy for three commonly used drugs, showing all 5 levels.**

Level	ATC Code and Description
**Metformin (A10BA02)**	
Level 1	A — Alimentary tract and metabolism
Level 2	A10 — Drugs used in diabetes
Level 3	A10B — Blood glucose lowering drugs, excl. insulins
Level 4	A10BA — Biguanides
Level 5	A10BA02 — Metformin

Continued on next page

Table 3 continued from previous page

**Level**	**ATC Code and Description**
**Paracetamol (Acetaminophen) (N02BE01)**	
Level 1	N — Nervous system
Level 2	N02 — Analgesics
Level 3	N02B — Other analgesics and antipyretics
Level 4	N02BE — Anilides
Level 5	N02BE01 — Paracetamol
**Omeprazole (A02BC01)**	
Level 1	A — Alimentary tract and metabolism
Level 2	A02 — Drugs for acid related disorders
Level 3	A02B — Drugs for peptic ulcer and gastro-oesophageal reflux disease
Level 4	A02BC — Proton pump inhibitors
Level 5	A02BC01 — Omeprazole

2 Dataset split strategy

2.1 Dataset split strategy in NDS

Each molecule was represented as a Morgan fingerprint with a radius of 2. Pairwise molecular dissimilarity between all 12,088 molecules was quantified using the Jaccard distance over the binary fingerprint vectors. Single-linkage agglomerative hierarchical clustering was then applied to the resulting pairwise distance matrix at a distance threshold of 0.50, yielding a total of 4,499 clusters. Following clustering, the clusters were randomly partitioned into a source domain and a target domain at an 8/2 ratio at the cluster level. Among the 2,088 labeled drugs, this random cluster-level assignment yielded 1,775 drugs in the source domain and 313 in the target domain, as reported in the main text. The unlabeled external molecules were distributed across both domains as part of their respective clusters, and unlabeled 1,775 of which in the target domain were leveraged for adversarial training in the domain adaptation module.

2.2 Quantitative Characterization of the Induced Domain Shift

2.2.1 Experimental Design and Quantification Methods

To quantitatively characterize the distribution shift induced by the cluster-based split, we conduct three analyses that capture discrepancies between the source and target domains from different perspectives: *distribution-level separability*, *structure-level divergence*, and *feature-space distance.*

(1) Distribution-level separability ($\mathcal{A}$-distance). The $\mathcal{A}$-distance measures the distinguishability between two domains [1]. If a classifier can reliably discriminate whether a sample originates from the source or target domain, the underlying distributions are considered different. $\mathcal{A}$-distance is defined as:

$$d_{\mathcal{A}} = 2(1 - 2\epsilon), \tag{1}$$

where ϵ denotes the error rate of a domain discriminator. A larger $\mathcal{A}$-distance indicates stronger domain discrepancy.

In this experiment, a Random Forest classifier with 50 trees and a maximum depth of 5 is trained as the domain discriminator. To avoid bias in the classifier caused by imbalanced domain sizes, we enforce a 1:1 class balance by randomly subsampling an equal number of samples from the source and target domains. The combined dataset is split into training and test subsets with a ratio of 7:3.

(2) Structure-level divergence (Scaffold overlap). To quantify structural differences, we compute the overlap of Bemis–Murcko scaffolds between the source and target domains [2]. Let S_{source} and S_{target} denote the sets of unique scaffolds present in the source and target domains, respectively. The scaffold overlap ratio is then defined as:

$$\text{Overlap} = \frac{|S_{\text{source}} \cap S_{\text{target}}|}{|S_{\text{target}}|}. \tag{2}$$

A lower overlap indicates fewer shared core molecular topologies and thus a stronger structural shift. To assess statistical significance, a chi-square test is performed based on the counts of overlapping and non-overlapping scaffolds.

(3) Feature-space distance (Nearest neighbor similarity). We further evaluate the discrepancy in chemical space by measuring the similarity between each target molecule and its nearest neighbor in the source domain [3]. Specifically, for each target molecule, we compute and record the maximum Jaccard similarity using the Morgan fingerprint to the source set, corresponding to its nearest neighbor in the source domain. The distribution of these similarities reflects the proximity between the two domains. A Mann-Whitney U test is conducted to evaluate the statistical significance of the difference between splitting strategies. A Mann-Whitney U is chosen because Tanimoto similarity scores typically exhibit non-Gaussian and skewed distributions, we employed this non-parametric test to compare median ranks, providing a robust statistical confirmation of feature-space isolation without assuming normality.

2.2.2 Results and Discussion

The quantitative results under random split and cluster-based split are summarized in Table 4.

Distribution-level separability. The $\mathcal{A}$-distance under random split is 0.0086, indicating that the source and target domains are nearly indistinguishable. In contrast, the value increases to 0.5698 under the cluster-based split, demonstrating that the two domains become substantially more separable. This result suggests that the proposed splitting strategy introduces a clear discrepancy in the underlying data distributions, making the domain discrimination task non-trivial.

Structure-level divergence. The scaffold overlap decreases from 34.06% under random split to 15.78% under cluster split, indicating a noticeable reduction in shared core molecular structures between the source and target domains. The chi-square test yields a p-value of 6.74×10^{-21}, confirming that this reduction is statistically significant. This demonstrates that the cluster-based split introduces a pronounced structural shift, where the target domain contains a larger proportion of structurally novel compounds.

Feature-space distance. The mean nearest-neighbor similarity decreases from 0.5762 under random split to 0.3717 under cluster split, indicating that target samples are substantially farther from the source domain in feature space. The Mann–Whitney U test yields a p-value of 3.85×10^{-213}, demonstrating that the difference in similarity distributions is highly significant. This result further confirms that the proposed split induces a marked shift in chemical space.

Table 4: Quantitative characterization of domain shift under different data splitting strategies.

**Metric**	**Random Split**	**Cluster Split**
$\mathcal{A}$-distance	0.0086	0.5698
Scaffold Overlap	34.06%	15.78%
Mean NN Similarity	0.5762	0.3717

3 Rationale for Truncation Limits of ADReCS IDs and ATC Codes

The truncation limits of 4 ADReCS IDs and 6 ATC codes are determined based on empirical data distribution analysis and experimental validation.

For ADReCS IDs, the cumulative distribution of ADR coverage across the dataset (Figure 1) shows that 100% of ADRs are associated with 4 or fewer ADReCS IDs, meaning a limit of 4 achieves complete coverage without any information loss.

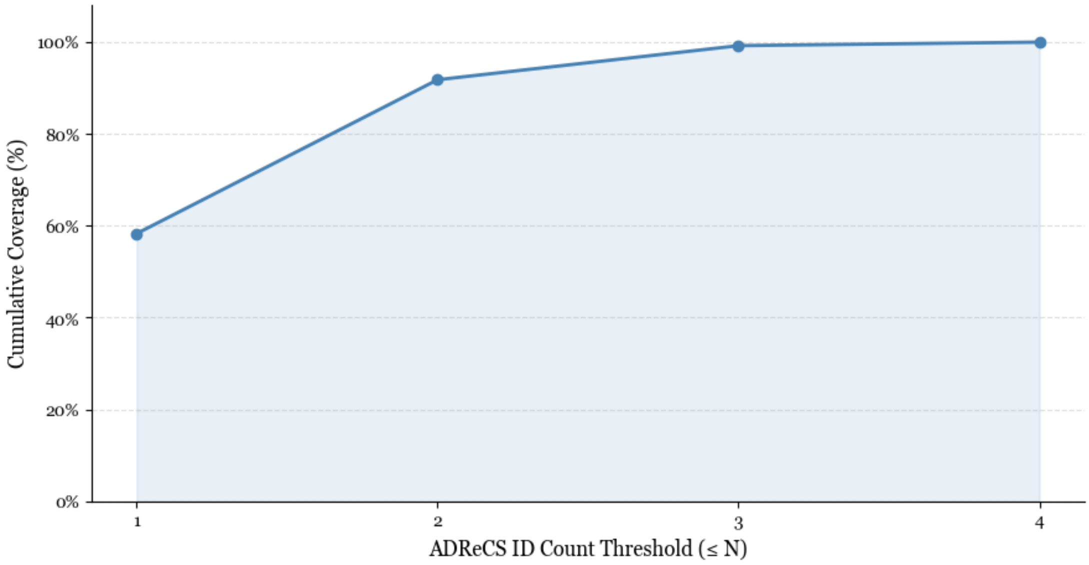

Figure 1: Cumulative ADR Coverage by ADReCS ID Count Threshold

For ATC codes, the cumulative distribution (Figure 2) shows that approximately 97% of drugs in the training set are associated with 6 or fewer ATC codes, so a limit of 6 ensures high information completeness while avoiding unnecessary zero-padding.

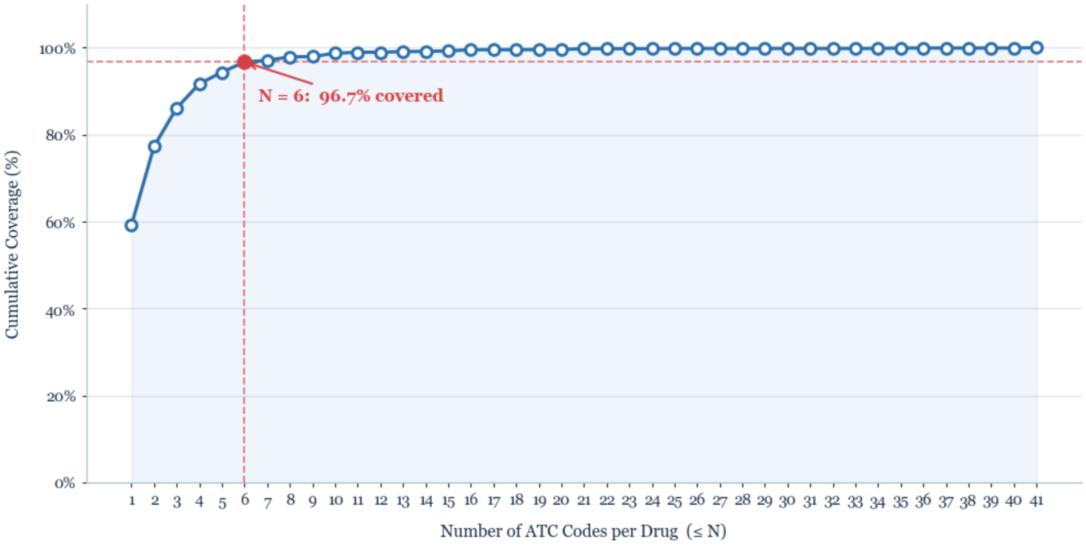

Figure 2: Cumulative Drug Coverage by ATC Code Threshold

To further validate this choice, we conducted experiments by varying the ATC code limit across three settings: 6, 10, and 20. As shown in Table 5, extending the limit from 6 to 10 (covering approximately 99% of training drugs) yielded only marginal improvements on several metrics, confirming that the limit of 6 is generally sufficient with negligible information loss. Notably, further increasing the limit to 20 led to a slight performance degradation, likely because excessive zero-padding dilutes effective feature representations and distorts the underlying statistical distribution, ultimately resulting in suboptimal model performance.

Table 5: **Performance comparison with different ATC code limits (mean ± std).**

Model	F1	ROC-AUC	PR-AUC	Precision	Recall	Accuracy
6 ATC codes	0.7753±0.0063	**0.8483±0.0021**	0.8498±0.0026	0.7707±0.0096	**0.7803±0.0090**	0.7753±0.0056
10 ATC codes	**0.7761±0.0058**	0.8480±0.0021	**0.8506±0.0027**	**0.7737±0.0101**	0.7791±0.0089	**0.7766±0.0051**
20 ATC codes	0.7744±0.0071	0.8477±0.0021	0.8476±0.0026	0.7708±0.0098	0.7781±0.0097	0.7740±0.0062

4 Baseline Details and Experimental Setup

4.1 Task-Specific Adaptations

PreciseADR was originally developed based on a heterogeneous graph neural network incorporating patients, diseases, drugs, and ADRs as graph nodes, with graph aggregation and patient node augmentation as key operations. Since our task does not involve patient-level information, patient nodes were replaced with drug ATC classification features represented as multi-hot encodings to maintain consistency with the feature space used in our framework. In addition, disease nodes were replaced with drug indication nodes due to their analogous functional roles. Consequently, the constructed heterogeneous graph consists of ATC categories, indications, drugs, and ADRs, forming a drug-centric graph structure. The original patient node augmentation operation was correspondingly adapted to drug node augmentation, while all remaining components, including the graph aggregation mechanism and prediction head, were implemented following the original design.

GCAP was originally proposed for predicting serious clinical outcomes associated with ADRs. The framework consists of drug and ADR representation modules, two auxiliary feature inputs (drug–ADR interaction and seriousness information), and an attention-based prediction pipeline. To adapt the model to our task setting, the auxiliary drug–ADR interaction feature was removed to avoid potential information leakage, and the seriousness-related feature was excluded because it is not applicable to ADR prediction. All other architectural components were retained without modification.

OrganADR was originally designed for DDI-induced ADR prediction and comprises a molecular feature module, an ADR association matrix, a GNN module, a GCN module, and an attention module. Since our task focuses on single-drug ADR prediction rather than drug–drug interactions, the GNN module responsible for integrating information from interacting drugs through knowledge graphs was removed. All remaining modules were preserved following the original implementation.

4.2 Environment and Training Protocol

To ensure a fair comparison, all models were trained and evaluated under the same experimental protocol, including data preprocessing, optimization settings, and evaluation procedures. A summary of the unified configurations is provided in Table 6.

4.3 Hyperparameter Configuration

Model-specific hyperparameters related to model capacity and optimization were retained according to their original configurations. For methods with publicly available implementations, including ML-CF, BiMPADR, GCAP, and OrganADR, the official codebases were directly adopted, while the remaining methods were re-implemented based on the descriptions provided in the original papers. For hyperparameters not explicitly reported, commonly used empirical settings were adopted. Detailed hyperparameter configurations are summarized in Table 7.

Table 6: Summary of the Unified Training and Evaluation Protocol

**Protocol Category**	**Unified Experimental Specifications and Configurations**
1 Data Engineering & Evaluation Protocol	
Dataset Splitting and Construction	All models were trained and evaluated on the same dataset as described in Section 2.2, with an identical random seed applied to negative sample generation to ensure reproducibility and comparability across all baselines.
Random Seeds	All models were evaluated under identical random seeds across 5 independent runs, with the final results reported as the mean ± standard deviation to eliminate statistical anomalies.
Evaluation Metrics	Metrics were consistently calculated using identical `scikit-learn` functions (including AUC-ROC, AUPRC, F1-score, precision, recall, and accuracy).
Classification Threshold	Set to a standard empirical threshold of 0.5.
2 Optimization & Control Protocol	
Maximum Training Epochs	Capped at a maximum of 200 epochs.
Early Stopping Criterion	Monitored via validation F1 with a uniform patience of 20 epochs to ensure full convergence without overfitting.
3 Hardware & Software Environment	
Hardware Specifications	All experiments were executed on the same machine equipped with an NVIDIA RTX 4090 GPU.
Software Backend	Standardized on PyTorch 2.5, and CUDA 13.0.

Table 7: Empirical Default Hyperparameters for Unspecified Baseline Variables

**Hyperparameter**	**Default Value**
Hidden Embedding Dimension (d)	128
Dropout Rate (p)	0.2
Weight Decay (L_2 Reg.)	1e-5
Learning Rate (lr)	1e-3
Batch Size	256
Activation Function	ReLU
Normalization Layer	BatchNorm
Optimization Algorithm	Adam

5 Statistical Significance of Performance Gains

To verify that our model's consistent improvements are statistically meaningful beyond run-to-run variability, we conduct rigorous paired t-tests against each baseline.

Experimental Procedure. All models were evaluated under five independent runs with different random seeds. For each seed, the same seed was applied to all models simultaneously, ensuring that any variation arising from data shuffling or weight initialization is shared across methods. This yields five matched score pairs $\{(s_k^{\text{ours}}, s_k^{\text{base}})\}_{k=1}^{5}$ for every combination of baseline and evaluation metric.

A two-tailed paired t-test was then applied to each pair. Denoting the per-seed differences as $d_k = s_k^{\text{ours}} - s_k^{\text{base}}$, the test statistic is

$$\bar{d} = \frac{1}{n}\sum_{k=1}^{n} d_k, \quad s_d = \sqrt{\frac{1}{n-1}\sum_{k=1}^{n}(d_k - \bar{d})^2}, \quad t = \frac{\bar{d}}{s_d/\sqrt{n}},$$

where $n = 5$ and t follows a t-distribution with df $= 4$ under the null hypothesis $H_0\colon \mu_d = 0$. This procedure is repeated for all 7 baselines $\times$ 6 metrics $=$ 42 pairs, and the resulting p-values are reported in Table 8 and Table 9.

Results and Discussion. The paired t-test results demonstrate the consistent superiority of our proposed model over all seven baselines across in both NDS and KDS. On KDS, virtually all 42 comparisons reach $p < 0.001$, with the sole exception being Precision against BiMPADR ($p = 0.9475$). On NDS, improvements in F1, ROC-AUC, PR-AUC, Recall, and Accuracy are overwhelmingly significant ($p < 0.001$) across all baselines, with only a small number of Precision comparisons (against GCAP and BiMPADR) and Recall against Kg-based falling outside the $\alpha = 0.05$ threshold. These exceptions are due to precision-recall trade-offs, in which our model attains a more favorable balance, leading to higher F1 scores. Overall, out of 84 paired comparisons, the vast majority achieve $p < 0.001$, providing strong statistical evidence that our model delivers robust and reliable improvements over the baselines.

Table 8: **Paired t-test p-values: our model vs. each baseline (NDS).**

Baseline	F1	ROC-AUC	PR-AUC	Precision	Recall	Accuracy
GCAP	6.11×10^{-5}	4.45×10^{-4}	6.81×10^{-5}	0.0528	1.20×10^{-4}	1.77×10^{-5}
OrganADR	1.69×10^{-4}	2.61×10^{-5}	1.38×10^{-5}	1.05×10^{-4}	0.0033	1.48×10^{-6}
PreciseADR	8.65×10^{-4}	5.52×10^{-6}	7.14×10^{-6}	0.0284	5.00×10^{-4}	9.02×10^{-4}
ML-CF	1.40×10^{-5}	4.24×10^{-5}	6.36×10^{-5}	0.0245	9.86×10^{-6}	7.72×10^{-5}
Image-CNN	1.65×10^{-4}	1.58×10^{-4}	0.0015	0.0372	4.67×10^{-5}	2.41×10^{-4}
BiMPADR	4.01×10^{-4}	4.69×10^{-5}	2.12×10^{-5}	0.0644	4.95×10^{-4}	2.33×10^{-5}
DrugBAN	2.45×10^{-4}	1.77×10^{-5}	1.01×10^{-5}	0.0123	8.76×10^{-4}	1.21×10^{-5}
Kg-based	0.0020	4.29×10^{-5}	4.28×10^{-5}	6.63×10^{-4}	0.8577	1.42×10^{-4}

Each cell reports the two-tailed p-value from a paired t-test over five independent runs (df = 4).
Color indicates significance: dark green $p < 0.001$, medium green $p < 0.01$, light green $p < 0.05$, light red $p \geq 0.05$.

Table 9: **Paired t-test p-values: our model vs. each baseline (KDS).**

Baseline	F1	ROC-AUC	PR-AUC	Precision	Recall	Accuracy
`GCAP`	1.32×10^{-7}	3.13×10^{-7}	2.81×10^{-7}	0.0221	6.05×10^{-5}	2.16×10^{-6}
`OrganADR`	8.38×10^{-7}	5.72×10^{-6}	1.35×10^{-6}	7.19×10^{-4}	1.15×10^{-5}	4.64×10^{-6}
`PreciseADR`	9.66×10^{-7}	4.39×10^{-6}	2.02×10^{-6}	0.0065	4.14×10^{-5}	5.69×10^{-6}
`ML-CF`	1.07×10^{-6}	4.38×10^{-6}	2.38×10^{-6}	5.75×10^{-4}	1.58×10^{-5}	9.07×10^{-6}
`Image-CNN`	2.11×10^{-8}	1.80×10^{-7}	5.25×10^{-7}	0.0071	4.45×10^{-7}	1.31×10^{-8}
`BiMPADR`	2.67×10^{-6}	5.76×10^{-6}	1.26×10^{-6}	0.9475	4.22×10^{-6}	2.59×10^{-5}
`Kg-based`	1.53×10^{-7}	8.42×10^{-7}	7.30×10^{-7}	7.34×10^{-5}	1.55×10^{-5}	8.86×10^{-6}

Each cell reports the two-tailed p-value from a paired t-test over five independent runs (df = 4).
Color indicates significance: dark green $p < 0.001$, medium green $p < 0.01$, light green $p < 0.05$, light red $p \geq 0.05$.

6 Computational Cost Analysis

We report the per-epoch training time and per-sample inference time for all models in Table 10, measured on a single NVIDIA 4090.

The baselines adopt two prediction paradigms. Methods that frame ADR prediction as multi-label classification (OrganADR, Image-CNN) output a prediction vector over all ADRs per drug, resulting in substantially fewer training instances and correspondingly shorter per-epoch training times. However, this efficiency comes at a representational cost, as these methods either forgo dedicated ADR embeddings or rely on coarse pooled aggregates, which limits their ability to capture fine-grained ADR semantics. Methods that support operating on individual drug–ADR pairs (GCAP, PreciseADR, ML-CF, BiMPADR, KG-based, and ours) support richer ADR representation learning at the cost of a larger training sample space. Notably, both paradigms utilize the same underlying drug–ADR association data.

Among pair-based methods, our model exhibits the longest per-epoch training time. Nevertheless, the gap relative to other pair-based methods is moderate, and the increased training cost is commensurate with the consistent performance gains reported in the main text. With respect to inference time, inference of our model for an individual sample takes on the order of milliseconds. As ADR prediction in pharmacovigilance is typically conducted as an offline screening task, the marginal increase in training time represents a justifiable trade-off, while the inference latency poses no practical barrier to deployment.

Table 10: **Computational cost comparison with baselines (NDS).**

Model	F1	Training Time (min/epoch)	Inference Time (ms/sample)
GCAP	0.6952±0.0099	4.0	0.47
OrganADR	0.6851±0.0139	0.2	0.31
PreciseADR	0.7179±0.0092	3.3	0.43
ML-CF	0.6550±0.0115	2.0	0.06
Image-CNN	0.6625±0.0159	0.1	0.17
BiMPADR	0.7157±0.0086	3.2	0.39
Kg-based	0.7282±0.0111	3.3	0.46
Ours	0.7753±0.0063	5.4	0.53

7 Ablation Study Results

7.1 NDS ablation results

Table 11: **Ablation study results in NDS (mean ± std).**

Model	F1	ROC-AUC	PR-AUC	Prec.	Rec.	Acc.
w/o ADR graph	0.7193±0.0111	0.8297±0.0036	0.8293±0.0052	0.8102±0.0119	0.6482±0.0124	0.7453±0.0056
w/o ADR hierarchical relations	0.7485±0.0089	0.8395±0.0041	0.8401±0.0044	0.7889±0.0108	0.7131±0.0098	0.7614±0.0059
w/o ADR co-occurrence	0.7467±0.0057	0.8486±0.0036	0.8461±0.0030	0.8016±0.0068	0.6995±0.0070	0.7638±0.0062
Homo-GCN	0.7264±0.0055	0.8160±0.0062	0.8112±0.0057	0.7753±0.0094	0.6843±0.0116	0.7434±0.0078
w/o intra	0.7312±0.0057	0.8312±0.0057	0.8319±0.0056	0.7880±0.0080	0.6812±0.0119	0.7502±0.0097
Random assignment	0.7211±0.0081	0.8237±0.0065	0.8251±0.0116	0.7741±0.0120	0.6816±0.0058	0.7457±0.0081
w/o inter	0.7592±0.0073	0.8438±0.0023	0.8446±0.0061	0.7789±0.0064	0.7399±0.0094	0.7677±0.0067
Single MLP	0.7204±0.0069	0.8150±0.0063	0.8175±0.0065	0.7726±0.0092	0.6759±0.0092	0.7389±0.0089
Single MLP-inter	0.7641±0.0062	0.8455±0.0024	0.8465±0.0029	0.7668±0.0089	0.7618±0.0087	0.7698±0.0062
w/o attention	0.7697±0.0061	0.8579±0.0038	0.8554±0.0029	0.7745±0.0057	0.7617±0.0075	0.7730±0.0045
w/o ATC	0.7603±0.0067	0.8363±0.0037	0.8341±0.0036	0.7599±0.0091	0.7643±0.0128	0.7615±0.0070
w/o ATC hierarchical relations	0.7605±0.0064	0.8378±0.0029	0.8352±0.0030	0.7604±0.0091	0.7664±0.0120	0.7604±0.0068
w/o ATC co-occurrence relations	0.7691±0.0055	0.8441±0.0030	0.8411±0.0029	0.7632±0.0089	0.7774±0.0104	0.7646±0.0068
w/o Molformer	0.7659±0.0070	0.8462±0.0037	0.8461±0.0036	0.7722±0.0075	0.7603±0.0103	0.7731±0.0071
w/o CDAN	0.7612±0.0043	0.8564±0.0021	0.8530±0.0022	0.8055±0.0056	0.7215±0.0074	0.7736±0.0056
Full Model	0.7753±0.0063	0.8483±0.0021	0.8498±0.0026	0.7707±0.0096	0.7803±0.0090	0.7753±0.0056

Discussion of the Divergence Between F1-score and AUC Metrics

In the CDAN ablation experiment, we observe that the inclusion of CDAN improves the F1-score, whereas the ranking-based metrics, ROC-AUC and PR-AUC, exhibit a slight decrease. The observed divergence between F1-score and AUC-based metrics may stem from the distinct evaluative focuses of threshold-dependent and ranking-based measures. Specifically, the F1-score assesses classification performance at a fixed threshold, while ROC-AUC and PR-AUC reflect the global ordering quality of prediction scores.

Within the CDAN framework, the domain discriminator incorporates classifier predictions as conditional information. Consequently, the feature extractor is adversarially incentivized to transform ambiguous representations (those near the 0.5 decision boundary) into class-discriminative patterns to minimize domain discrepancy. While this process enhances the classification of borderline samples—thereby boosting the F1-score—it may simultaneously introduce subtle perturbations to the global score distribution, which could lead to small decreases in ranking-based metrics.

One possible reason why this phenomenon occurs is that ADR datasets tend to contain inherent noise and reporting biases, particularly when incorporating data from systems like FAERS [3, 4, 5]. Under such conditions, the domain alignment process requires more intensive feature-space adjustments to separate difficult samples near the boundary, thereby disturbing the ordering among samples. Furthermore, since the w/o CDAN variant already achieves high ROC-AUC and PR-AUC values, the ranking structure is largely saturated. In this setting, improving hard borderline classification may introduce slight ranking inconsistencies, leading to improved F1-score at the expense of minor decreases in AUC-based metrics.

7.2 KDS ablation results

Table 12: **Ablation study results in KDS (mean ± std).**

Model	F1	ROC-AUC	PR-AUC	Prec.	Rec.	Acc.
w/o ADR graph	0.8215±0.0013	0.9017±0.0015	0.8816±0.0019	0.7873±0.0033	0.8599±0.0040	0.8254±0.0014
w/o ADR hierarchical relations	0.8393±0.0010	0.9211±0.0009	0.9042±0.0010	0.8069±0.0027	0.8755±0.0030	0.8434±0.0010
w/o ADR co-occurrence	0.8499±0.0006	0.9284±0.0004	0.9118±0.0005	0.8146±0.0029	0.8895±0.0026	0.8532±0.0005
Homo-GCN	0.8420±0.0006	0.9225±0.0006	0.9060±0.0005	0.8060±0.0034	0.8823±0.0033	0.8452±0.0006
w/o intra	0.8455±0.0007	0.9270±0.0006	0.9110±0.0007	0.8046±0.0032	0.8918±0.0040	0.8477±0.0008
Random assignment	0.8408±0.0009	0.9217±0.0010	0.9047±0.0012	0.8023±0.0030	0.8843±0.0039	0.8424±0.0010
w/o inter	0.8507±0.0010	0.9298±0.0011	0.9134±0.0012	0.8158±0.0027	0.8897±0.0042	0.8540±0.0009
Single MLP	0.8439±0.0010	0.9252±0.0012	0.9081±0.0013	0.8057±0.0030	0.8869±0.0040	0.8466±0.0011
Single MLP-inter	0.8530±0.0007	0.9314±0.0004	0.9153±0.0004	0.8194±0.0025	0.8904±0.0036	0.8563±0.0006
w/o attention	0.8541±0.0005	0.9335±0.0005	0.9185±0.0006	0.8275±0.0023	0.8834±0.0030	0.8589±0.0007
Full Model	0.8555±0.0005	0.9330±0.0003	0.9172±0.0005	0.8171±0.0023	0.8971±0.0031	0.8581±0.0004

8 Performance Comparison with Baselines on Different ADR Rarity Buckets

8.1 Main experiments

The calculation formulas for the Matthews Correlation Coefficient (MCC) and Balanced Accuracy in the following experiments are as follows:

$$\text{MCC} = \frac{TP \cdot TN - FP \cdot FN}{\sqrt{(TP+FP)(TP+FN)(TN+FP)(TN+FN)}}, \tag{3}$$

$$\text{Balanced Accuracy} = \frac{1}{2}\left(\frac{TP}{TP+FN} + \frac{TN}{TN+FP}\right), \tag{4}$$

where TP, TN, FP, and FN represent the number of true positives, true negatives, false positives, and false negatives, respectively.

Table 13: **Performance comparison with baseline models on different ADR rarity buckets (mean ± std).** From top to bottom, the five blocks correspond to the very common, common high, common medium, common low, and uncommon buckets, respectively.

Model	NDS (1) ROC-AUC	NDS (1) MCC	NDS (1) Balanced accuracy	KDS (2) ROC-AUC	KDS (2) MCC	KDS (2) Balanced accuracy
BiMPADR	0.6616±0.0031	0.0771±0.0026	0.5155±0.0026	0.8406±0.0013	0.4720±0.0010	0.6962±0.0013
PreciseADR	0.6901±0.0030	0.1050±0.0025	0.5303±0.0022	0.8299±0.0018	0.4635±0.0014	0.6869±0.0013
Kg-based(1)/ GCAP(2)	0.6881±0.0038	0.1021±0.0022	0.5267±0.0014	0.8256±0.0152	0.4567±0.0034	0.6777±0.0013
Ours	**0.7451±0.0036**	**0.3046±0.0030**	**0.6298±0.0035**	**0.8724±0.0008**	**0.5609±0.0010**	**0.7417±0.0008**
BiMPADR	0.5684±0.0033	0.0880±0.0003	0.5440±0.0016	0.8592±0.0012	0.5629±0.0012	0.7814±0.0013
PreciseADR	0.6399±0.0030	0.0828±0.0006	0.5623±0.0020	0.8519±0.0015	0.5542±0.0017	0.7765±0.0017
Kg-based(1)/ GCAP(2)	0.6421±0.0033	0.0730±0.0012	0.5677±0.0014	0.8420±0.0014	0.5421±0.0018	0.7698±0.0016
Ours	**0.7030±0.0027**	**0.2865±0.0030**	**0.6427±0.0029**	**0.8990±0.0007**	**0.6440±0.0009**	**0.8219±0.0009**
BiMPADR	0.5827±0.0045	0.0389±0.0016	0.5068±0.0006	0.8780±0.0010	0.5629±0.0011	0.7556±0.0010
PreciseADR	0.6403±0.0037	0.0999±0.0003	0.5201±0.0003	0.8699±0.0013	0.5325±0.0013	0.7541±0.0013
Kg-based(1)/ GCAP(2)	0.6498±0.0033	0.1021±0.0019	0.5205±0.0014	0.8601±0.0016	0.5256±0.0016	0.7156±0.0015
Ours	**0.7339±0.0025**	**0.2748±0.0028**	**0.6113±0.0020**	**0.9144±0.0009**	**0.6444±0.0008**	**0.8265±0.0008**
BiMPADR	0.5553±0.0021	-0.0077±0.0006	0.4994±0.0003	0.8772±0.0013	0.3898±0.0013	0.6383±0.0015
PreciseADR	0.6067±0.0031	0.0483±0.0013	0.5101±0.0013	0.8759±0.0017	0.4649±0.0017	0.6932±0.0017
Kg-based(1)/ GCAP(2)	0.6125±0.0033	0.0489±0.0012	0.5111±0.0024	0.8689±0.0012	0.4578±0.0015	0.6854±0.0015
Ours	**0.7309±0.0033**	**0.1815±0.0031**	**0.5548±0.0034**	**0.9215±0.0009**	**0.6081±0.0009**	**0.7924±0.0008**
BiMPADR	0.4989±0.0002	-0.0040±0.0003	0.4996±0.0004	0.8050±0.0014	0.2490±0.0016	0.5480±0.0016
PreciseADR	0.5689±0.0011	0.0000±0.0001	0.5000±0.0002	0.8085±0.0016	0.3742±0.0017	0.6245±0.0017
Kg-based(1)/ GCAP(2)	0.5758±0.0013	0.0001±0.0002	0.4999±0.0006	0.8098±0.0019	0.3746±0.0017	0.6257±0.0019
Ours	**0.6984±0.0016**	**0.1453±0.0018**	**0.5358±0.0005**	**0.8708±0.0013**	**0.5221±0.0015**	**0.7222±0.0010**

8.2 Performance on Rare ADRs

Under the threshold of $\geq$ 10 drugs, 3,114 ADRs were retained. Following the same data construction pipeline described in **Section 2.2.2 Dataset partitioning and construction**, the datasets were reconstructed accordingly. For the NDS, training and test sets were formed by pairing each drug with all ADRs, yielding 1,775×3,114 and 313x3114 candidate pairs, respectively. After downsampling negatives to balance classes, 244,290 training pairs and 80,312 test pairs were obtained. For the KDS, a total of 369,117 pairs were obtained after negative downsampling, which were then randomly split into training (80%) and test (20%) sets.

Table 14: **Performance comparison with baseline models on different ADR rarity buckets (≥10 drugs threshold) (mean ± std).** From top to bottom, the six blocks correspond to the very common, common high, common medium, common low, uncommon, and rare buckets, respectively.

	NDS (1)			KDS (2)		
Model	ROC-AUC	MCC	Balanced accuracy	ROC-AUC	MCC	Balanced accuracy
BiMPADR	0.6441±0.0030	0.0748±0.0025	0.5013±0.0023	0.8187±0.0012	0.4591±0.0010	0.6784±0.0014
PreciseADR	0.6712±0.0025	0.0919±0.0023	0.5163±0.0018	0.8085±0.0019	0.4502±0.0014	0.6693±0.0012
Kg-based(1)/ GCAP(2)	0.6694±0.0037	0.0991±0.0021	0.5129±0.0012	0.8041±0.0016	0.4438±0.0028	0.6601±0.0016
Ours	**0.7241±0.0031**	**0.2758±0.0029**	**0.6023±0.0034**	**0.8502±0.0008**	**0.5447±0.0012**	**0.7221±0.0010**
BiMPADR	0.5521±0.0031	0.0854±0.0004	0.5289±0.0014	0.8371±0.0011	0.5481±0.0013	0.7614±0.0011
PreciseADR	0.6224±0.0026	0.0805±0.0007	0.5472±0.0020	0.8298±0.0016	0.5393±0.0015	0.7563±0.0018
Kg-based(1)/ GCAP(2)	0.6244±0.0033	0.0709±0.0010	0.5523±0.0015	0.8201±0.0013	0.5274±0.0019	0.7498±0.0013
Ours	**0.6831±0.0023**	**0.2631±0.0025**	**0.6248±0.0028**	**0.8768±0.0009**	**0.6263±0.0009**	**0.8009±0.0008**
BiMPADR	0.5661±0.0043	0.0378±0.0014	0.5011±0.0003	0.8559±0.0012	0.5481±0.0009	0.7364±0.0002
PreciseADR	0.6225±0.0035	0.0970±0.0004	0.5061±0.0003	0.8474±0.0011	0.5176±0.0003	0.7349±0.0011
Kg-based(1)/ GCAP(2)	0.6318±0.0028	0.0992±0.0018	0.5063±0.0012	0.8378±0.0017	0.5109±0.0014	0.6974±0.0016
Ours	**0.7131±0.0025**	**0.2569±0.0023**	**0.5938±0.0021**	**0.8914±0.0010**	**0.6267±0.0008**	**0.8058±0.0009**
BiMPADR	0.5391±0.0019	0.0047±0.0004	0.4999±0.0002	0.8551±0.0014	0.3793±0.0005	0.6219±0.0016
PreciseADR	0.5897±0.0030	0.0369±0.0011	0.5011±0.0013	0.8537±0.0015	0.4526±0.0018	0.6751±0.0015
Kg-based(1)/ GCAP(2)	0.5953±0.0028	0.0375±0.0008	0.5011±0.0022	0.8469±0.0011	0.4456±0.0016	0.6677±0.0013
Ours	**0.7099±0.0032**	**0.1692±0.0030**	**0.5393±0.0029**	**0.8984±0.0008**	**0.5913±0.0011**	**0.7719±0.0007**
BiMPADR	0.4989±0.0002	0.0004±0.0001	0.4996±0.0004	0.7799±0.0014	0.2423±0.0014	0.5337±0.0017
PreciseADR	0.5527±0.0012	0.0000±0.0001	0.5001±0.0001	0.7873±0.0013	0.3641±0.0018	0.6083±0.0015
Kg-based(1)/ GCAP(2)	0.5594±0.0012	0.0001±0.0001	0.4996±0.0002	0.7887±0.0020	0.3645±0.0015	0.6094±0.0018
Ours	**0.6789±0.0016**	**0.1391±0.0016**	**0.5213±0.0005**	**0.8481±0.0011**	**0.5081±0.0016**	**0.7034±0.0009**
BiMPADR	0.5005±0.0003	0.0003±0.0001	0.5001±0.0001	0.7134±0.0008	0.1467±0.0020	0.5000±0.0001
PreciseADR	0.5213±0.0002	0.0001±0.0001	0.5003±0.0002	0.7323±0.0010	0.2596±0.0015	0.5665±0.0014
Kg-based(1)/ GCAP(2)	0.5210±0.0003	0.0002±0.0001	0.4998±0.0002	0.7211±0.0015	0.2503±0.0024	0.5633±0.0017
Ours	**0.6069±0.0011**	**0.0009±0.0001**	**0.5002±0.0001**	**0.7923±0.0005**	**0.4071±0.0011**	**0.6691±0.0011**

9 Data sources and preprocessing of SIDER

9.1 Dataset Sources

Drug Information: We sourced drug names from the SIDER database and retrieved their corresponding SMILES strings from DrugBank. ATC classification codes were obtained directly from SIDER.

ADR Information: We utilized ADR PT from the SIDER database and mapped them to the ADReCS database to obtain standardized hierarchical identifiers (formatted as `xx.xx.xx.xxx`). ADRs associated with fewer than 10 drugs (approximately 1% of the total drug count) were excluded. The final preprocessed dataset comprises 1,004 drugs (hereafter referred to as ADR drugs) and 1,214 unique ADRs.

9.2 Dataset partitioning and construction

KDS dataset. Following the same procedure as described in Section **??**, we introduced a distribution shift by clustering the 1,004 ADR drugs with external drugs and partitioning the resulting clusters into a source domain and a target domain. This procedure yielded 836 ADR drugs in the source domain for training and 168 ADR drugs in the target domain for testing.

For both the training and testing sets, drug–ADR pairs were first constructed by combining the respective drugs with the ADR set. Among these pairs, the known drug–ADR associations provided in the dataset were treated as positive samples. Since SIDER only contains positive associations, all remaining unobserved drug–ADR pairs were regarded as negative candidates. An equal number of negative samples were randomly selected to construct a 1:1 positive-to-negative ratio for both training and testing.

NDS dataset. All 1,004 ADR drugs were paired with the 1,214 ADRs to form a candidate pool. Known drug–ADR pairs in the dataset were treated as positive samples, and an equal number of negative samples were randomly selected from the remaining unobserved pairs. The resulting integrated pool of positive and negative samples was then randomly partitioned into training and testing sets using an 8:2 ratio.

10 Results of Robustness Analysis

Table 15: **Results of robustness analysis of our model and the two strongest baselines under varying degrees of training data sparsity in NDS (mean ± std).**

Model	F1	ROC-AUC	PR-AUC	Precision	Recall	Accuracy
Ours (Full Data)	0.7753±0.0063	0.8483±0.0021	0.8498±0.0026	0.7707±0.0096	0.7803±0.0090	0.7753±0.0056
10% drug removed	0.7528±0.0068	0.8234±0.0024	0.8215±0.0027	0.6582±0.0105	0.8791±0.0128	0.7402±0.0068
20% drug removed	0.7415±0.0079	0.8115±0.0028	0.8092±0.0029	0.6438±0.0118	0.8735±0.0142	0.7285±0.0082
30% drug removed	0.7373±0.0052	0.8042±0.0032	0.8066±0.0035	0.6354±0.0092	0.8784±0.0104	0.7241±0.0059
40% drug removed	0.7091±0.0114	0.7721±0.0034	0.7738±0.0038	0.6125±0.0142	0.8420±0.0185	0.6912±0.0105
PreciseADR (Full Data)	0.7179±0.0092	0.7791±0.0054	0.7742±0.0043	0.7478±0.0114	0.6913±0.0148	0.7287±0.0084
10% drug removed	0.6443±0.0093	0.7434±0.0051	0.6911±0.0058	0.7002±0.0115	0.5583±0.0121	0.6330±0.0051
20% drug removed	0.6379±0.0108	0.7328±0.0042	0.6854±0.0055	0.6821±0.0132	0.5990±0.0154	0.6295±0.0072
30% drug removed	0.6201±0.0125	0.7185±0.0065	0.6720±0.0058	0.6554±0.0168	0.5885±0.0192	0.6184±0.0095
40% drug removed	0.6115±0.0114	0.7052±0.0049	0.6598±0.0061	0.6412±0.0145	0.5844±0.0178	0.6092±0.0112
Kg-based (Full Data)	0.7282±0.0111	0.7904±0.0067	0.7855±0.0067	0.6893±0.0142	0.7780±0.0187	0.7189±0.0086
10% drug removed	0.6917±0.0095	0.7581±0.0058	0.7523±0.0064	0.6554±0.0155	0.7324±0.0185	0.6825±0.0102
20% drug removed	0.6754±0.0128	0.7324±0.0072	0.7255±0.0088	0.6392±0.0172	0.7162±0.0205	0.6672±0.0128
30% drug removed	0.6641±0.0105	0.7218±0.0085	0.7132±0.0082	0.6278±0.0178	0.7051±0.0212	0.6508±0.0115
40% drug removed	0.6547±0.0122	0.7015±0.0105	0.6938±0.0118	0.6188±0.0145	0.6942±0.0228	0.6452±0.0134

Table 16: **Results of robustness analysis of our model and the two strongest baselines under varying degrees of training data sparsity in KDS (mean ± std).**

Model	F1	ROC-AUC	PR-AUC	Precision	Recall	Accuracy
Ours (Full Data)	0.8555±0.0005	0.9330±0.0003	0.9172±0.0005	0.8171±0.0023	0.8971±0.0031	0.8581±0.0004
10% data removed	0.8432±0.0012	0.9285±0.0008	0.9105±0.0009	0.7895±0.0045	0.9040±0.0048	0.8391±0.0028
20% data removed	0.8389±0.0018	0.9248±0.0011	0.9058±0.0013	0.7791±0.0048	0.9080±0.0054	0.8312±0.0031
30% data removed	0.8350±0.0025	0.9215±0.0015	0.9018±0.0017	0.7702±0.0054	0.9115±0.0064	0.8248±0.0037
40% data removed	0.8305±0.0031	0.9178±0.0019	0.8972±0.0021	0.7598±0.0063	0.9165±0.0073	0.8165±0.0045
PreciseADR (Full Data)	0.8178±0.0013	0.9043±0.0020	0.8802±0.0018	0.8051±0.0045	0.8306±0.0062	0.8268±0.0020
10% data removed	0.7900±0.0028	0.8935±0.0016	0.8715±0.0018	0.7612±0.0051	0.8213±0.0059	0.7952±0.0043
20% data removed	0.7932±0.0035	0.8891±0.0020	0.8658±0.0022	0.7548±0.0060	0.8351±0.0068	0.7891±0.0049
30% data removed	0.7921±0.0042	0.8852±0.0024	0.8608±0.0026	0.7491±0.0069	0.8402±0.0079	0.7825±0.0055
40% data removed	0.7859±0.0048	0.8805±0.0028	0.8545±0.0030	0.7385±0.0073	0.8398±0.0086	0.7735±0.0059
BiMPADR (Full Data)	0.8155±0.0021	0.9079±0.0016	0.8896±0.0011	0.8173±0.0049	0.8140±0.0058	0.8277±0.0034
10% data removed	0.7919±0.0027	0.9012±0.0015	0.8815±0.0017	0.7889±0.0046	0.7952±0.0051	0.8038±0.0031
20% data removed	0.7815±0.0033	0.8965±0.0019	0.8755±0.0021	0.7735±0.0056	0.7898±0.0064	0.7912±0.0044
30% data removed	0.7778±0.0039	0.8925±0.0023	0.8705±0.0025	0.7648±0.0068	0.7912±0.0078	0.7851±0.0054
40% data removed	0.7705±0.0045	0.8878±0.0027	0.8645±0.0029	0.7512±0.0074	0.7905±0.0084	0.7761±0.0058

11 Case Studies

Case Study 1: Taxonomic Anchoring via ATC R-GCN Supports Prediction for Novel Drugs

Praziquantel is an anthelmintic drug primarily used to treat schistosomiasis. Relative to the majority of compounds in our training set, it has limited overlap in molecular scaffolds, known targets and indications. Such sparsity poses challenges for existing prediction methods, which depend on exact overlap in fine-grained biomedical annotations to establish meaningful associations with known compounds. In contrast, our model successfully predicted this ADR by anchoring Praziquantel within the ATC hierarchy, where coarser taxonomic relationships enable knowledge transfer even in the absence of precise feature-level correspondences, and hierarchical message passing further enriches representations across multiple levels of granularity — broader ancestral nodes contribute shared class-level immunological context, while deeper neighboring nodes reinforce more specific signatures.

Case Study 2: ADR Co-occurrence Edges Recover Linked Adverse Events

Simvastatin, a widely prescribed statin for hypercholesterolemia, is known to cause both myopathy and rhabdomyolysis — the latter representing a severe manifestation of statin-induced muscle injury. While myopathy carries a strong pharmacovigilance signal and is correctly predicted by the baseline model, rhabdomyolysis is comparatively rare in isolation, causing the baseline to assign it a substantially lower confidence score that falls below the prediction threshold. Our model recovers this missing prediction through ADR co-occurrence edges: because myopathy and rhabdomyolysis co-occur at high frequency across the training corpus, a strong edge connects the two ADR nodes in the graph. The robust signal propagated to the myopathy node flows across this edge, providing sufficient evidence to activate the rhabdomyolysis prediction. This illustrates how co-occurrence edges serve not merely as statistical correlations, but as biologically grounded propagation channels that generalise from well-evidenced ADRs to their mechanistically coupled counterparts.

Case Study 3: Hierarchy Anchors Rare ADR Representations Against Data Sparsity

Mexiletine is a sodium channel blocker used in the management of ventricular arrhythmias, whose ADR profile is dominated by cardiac and gastrointestinal events. Paraesthesia, though mechanistically plausible given mexiletine's peripheral sodium channel activity, is an infrequently reported ADR that yields only a modest signal in pharmacovigilance databases, and the baseline fail to confidently associate it with the drug. Our model recovers this prediction by anchoring the paraesthesia node within its MedDRA taxonomic family. Through hierarchical message passing, the node accumulates contextual signal from its more frequently reported relatives and broader ancestor nodes spanning neurological signs and symptoms and peripheral neuropathies. This rich neighbourhood stabilises the otherwise sparse representation of paraesthesia, yielding more stable and noise-resilient representations that enable confident prediction.

Additional Case Studies on Intra-Expert Router Weight Distribution

The second drug, Apomorphine hydrochloride [6], is classified under N04BC07 (Parkinson's

disease) and G04BE07 (erectile dysfunction). For nausea, the router assigned full weight to the N category, consistent with clinical evidence: nausea is common when apomorphine is used for advanced Parkinson's disease (subcutaneous route, typically higher dose) [7], but is infrequent and mild in erectile dysfunction treatment [8]. The third drug, Misoprostol, is associated with ATC codes G02AD06 (uterotonic use), A02BB01 (peptic ulcer prevention and treatment), and M01AE56 (in combination with naproxen for arthritis management) [6, 9]. For urticaria, the router assigned very high confidence to the M category (M: 0.9971), which is empirically supported: no urticaria-related reports are found for misoprostol alone under G02AD06 or A02BB01 in the SIDER database [10], whereas Naproxen is known to cause urticaria in postmarketing data [10]. Overall, the router provides an interpretable gating mechanism by assigning differential weights that reflect the most likely source of a given ADR and guide the selection of the most appropriate expert.

12 Impact of Hierarchical Granularity

We investigate how the granularity of hierarchical information affects model performance under the two settings by comparing predictions using ATC or ADR embeddings at different hierarchy levels. For ATC, we use fifth-, fourth-, and third-level embeddings, and for ADR, we use fourth-, third-, and second-level embeddings. Results are shown in Figure 3 (see Table 17, 18, 19 for details).

It is demonstrated in Figure 3(1a) that using coarser ATC levels leads to a decrease in performance, and similar patterns (Figure 3(1b) and (2)) are also observed for ADR hierarchies, indicating that finer-grained representations are beneficial for predictions. This can be explained by the information content of the embeddings: high-level nodes mainly capture blurred overall category information, lacking specific distinctions between individual drugs or ADRs. In contrast, finer-grained embeddings retain the aggregated context from higher-level nodes while also providing detailed distinctions for each drug or ADR. This richer information enables the model to capture commonalities while still differentiating drugs or ADRs, leading to more accurate predictions.

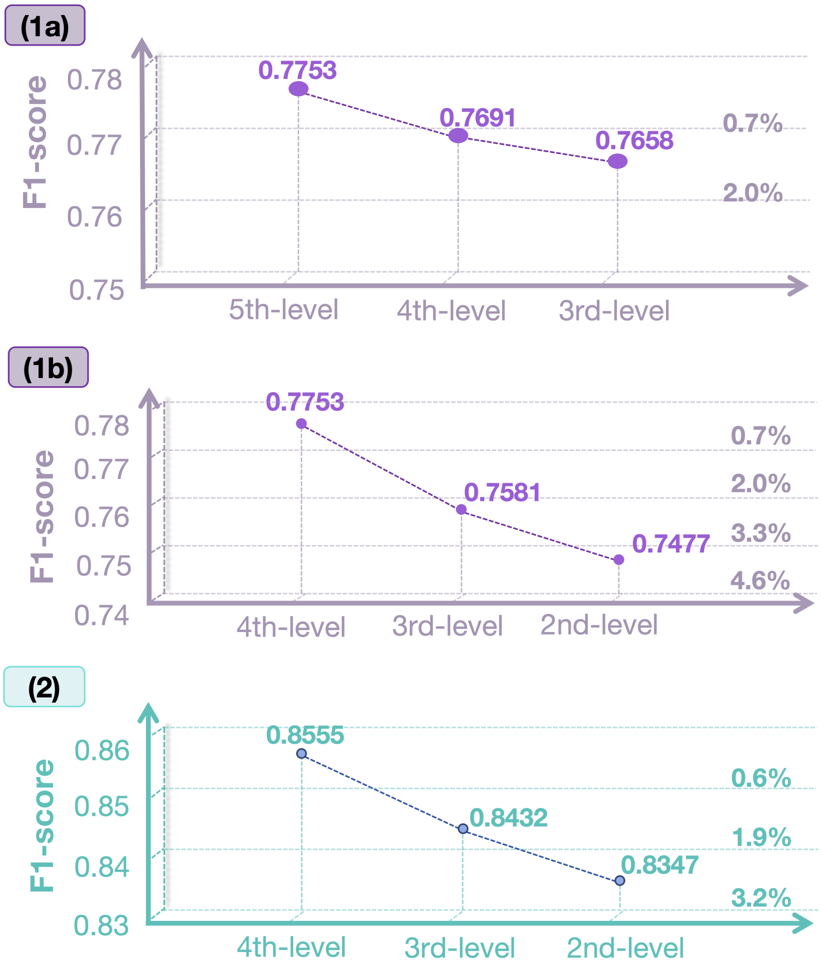

Figure 3: **Effect of hierarchy granularity on F1.** (1) NDS with (a) ATC embeddings, and (b) ADR embeddings at different levels. (2) KDS with ADR embeddings at different levels.

Table 17: **Results using ADR embeddings at different hierarchical levels under the standard scenario(5th, 4th, and 3rd) in NDS (mean ± std).**

Model	F1	ROC-AUC	PR-AUC	Precision	Recall	Accuracy
Fifth-level	0.7753±0.0083	0.8483±0.0021	0.8498±0.0026	0.7707±0.0096	0.7803±0.0090	0.7753±0.0056
Fourth-level	0.7691±0.0095	0.8399±0.0054	0.8468±0.0042	0.7800±0.0102	0.7581±0.0245	0.7701±0.0070
Third-level	0.7658±0.0130	0.8383±0.0065	0.8448±0.0056	0.7942±0.0133	0.7461±0.0264	0.7659±0.0064

Table 18: **Results using ADR embeddings at different hierarchical levels (4th, 3rd, and 2nd) in NDS (mean ± std).**

Model	F1	ROC-AUC	PR-AUC	Precision	Recall	Accuracy
Fourth-level	0.7753±0.0063	0.8483±0.0021	0.8498±0.0026	0.7707±0.0096	0.7803±0.0090	0.7753±0.0056
Third-level	0.7581±0.0075	0.8311±0.0032	0.8326±0.0039	0.7534±0.0145	0.7628±0.0136	0.7490±0.0088
Second-level	0.7477±0.0080	0.8207±0.0043	0.8222±0.0034	0.7431±0.0138	0.7524±0.0186	0.7467±0.0080

Table 19: **Results using ADR embeddings at different hierarchical levels (4th, 3rd, and 2nd) in KDS (mean ± std).**

Model	F1	ROC-AUC	PR-AUC	Precision	Recall	Accuracy
Fourth-level	0.8555±0.0005	0.9330±0.0003	0.9172±0.0005	0.8171±0.0023	0.8971±0.0031	0.8581±0.0004
Third-level	0.8432±0.0004	0.9301±0.0004	0.9154±0.0005	0.8035±0.0025	0.8863±0.0031	0.8503±0.0005
Second-level	0.8347±0.0005	0.9267±0.0007	0.9107±0.0005	0.7996±0.0031	0.8689±0.0039	0.8423±0.0006

13 Parameter Sensitivity Analysis

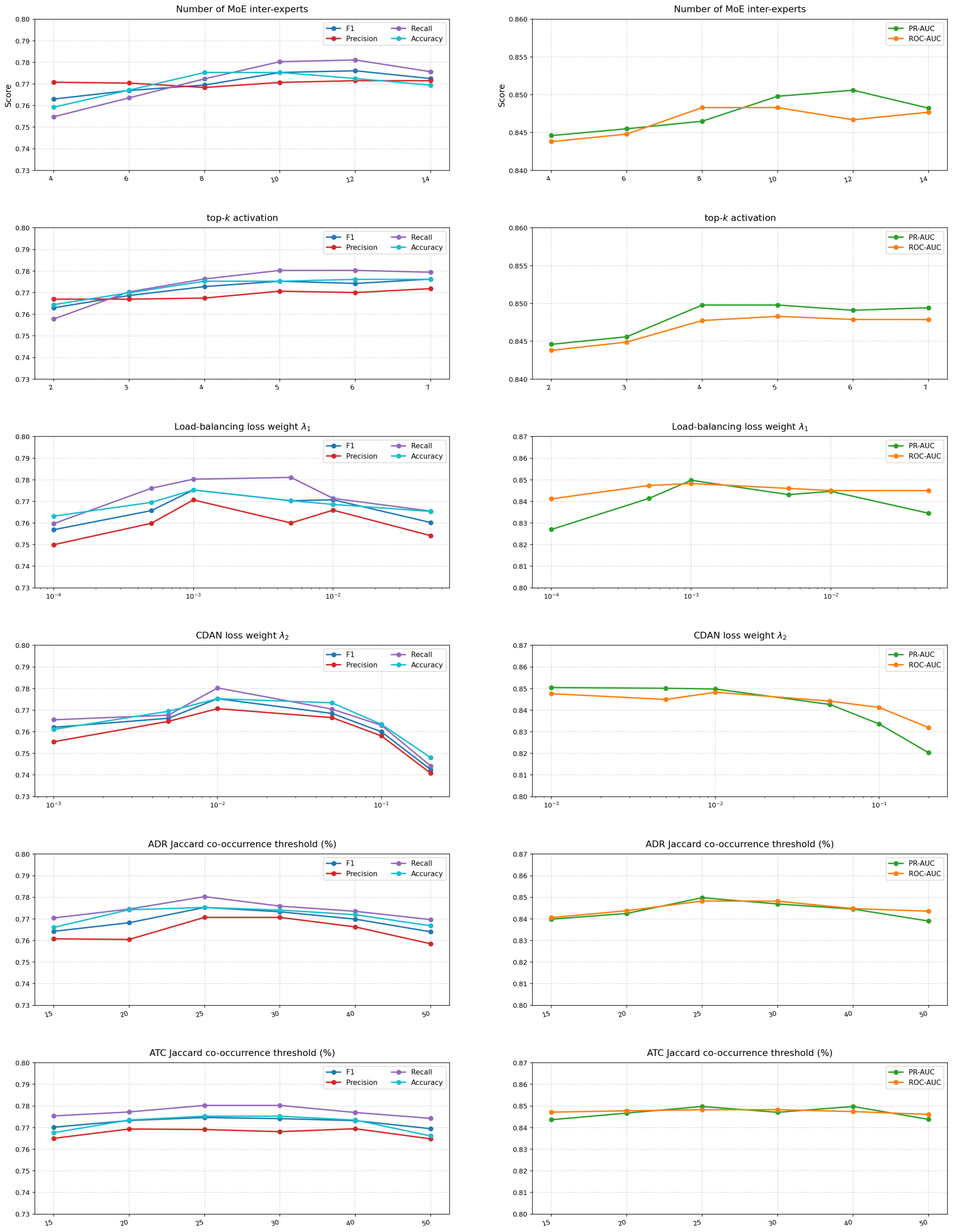

Figure 4: **Sensitivity analysis of the proposed model in NDS with respect to key hyperparameters (the number of MoE inter-experts, the top-k activation, the load-balancing loss weight λ_1, the CDAN loss weight λ_2, and the ADR and ATC Jaccard co-occurrence threshold).**

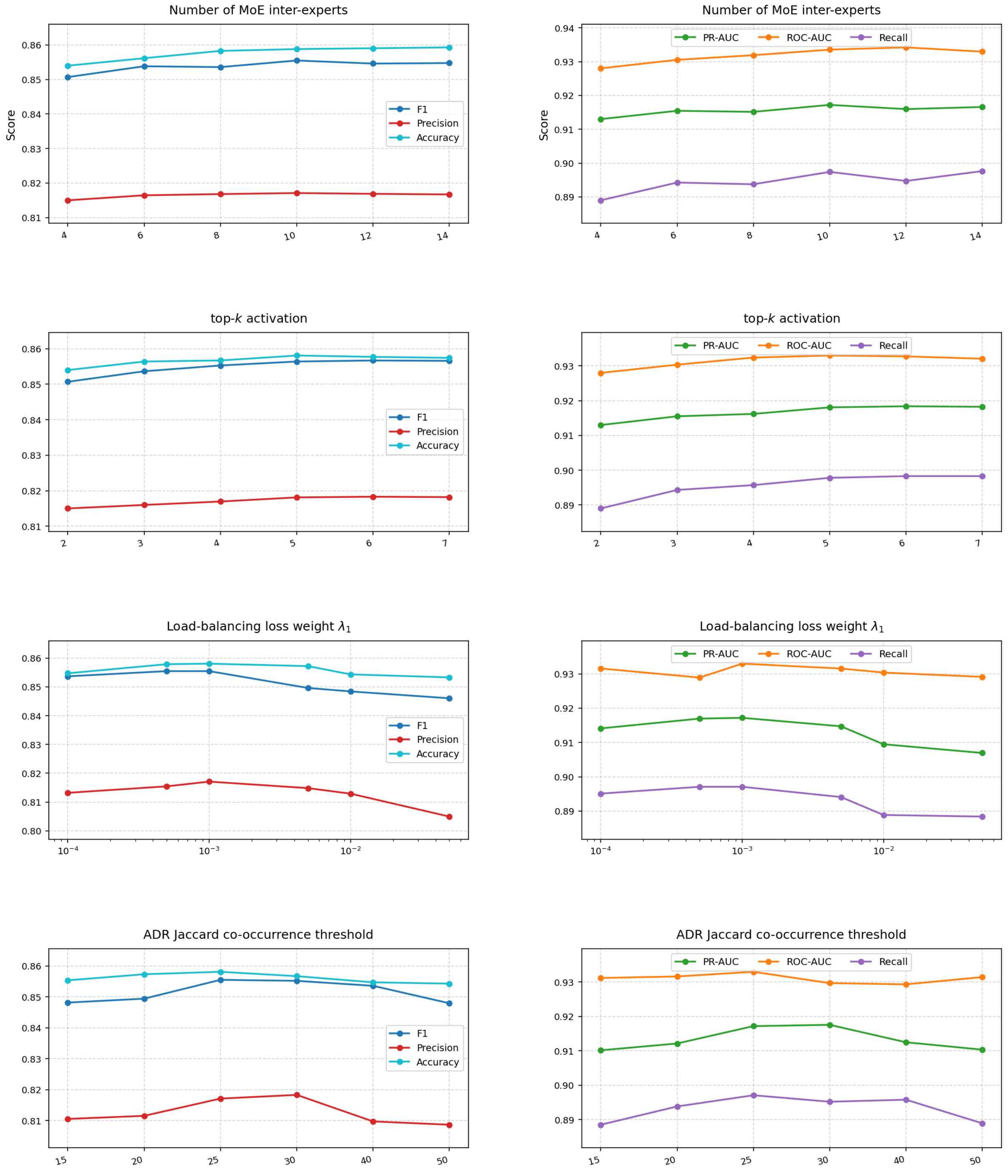

Figure 5: **Sensitivity analysis of the proposed model in KDS with respect to key hyperparameters (the number of MoE inter-experts, the top-k activation, the load-balancing loss weight λ_1, and the ADR Jaccard co-occurrence threshold).**

14 Hyperparameter Settings of the proposed model

Table 20: Hyperparameter Settings

Category	Hyperparameter	Value
**Training Configuration**	Optimizer	Adam
	Learning Rate	0.0002
	Weight Decay	1×10^{-5}
	Batch Size (Train / Test)	256 / 256
	Maximum number of training epochs	200
**Learning Rate Scheduler**	Scheduler Type	ReduceLROnPlateau
	Monitor Metric	Internal Validation Loss
	Decay Factor	0.5
	Patience (epochs)	6
**Early Stopping**	Patience (epochs)	20
	Monitor Metric	Internal Validation F1-score
**R-GCN (ADR & ATC Graphs)**	Number of Relation Types	4
	Number of Layers	3
**Dual-MoE**	Number of Intra-Experts (ATC L2)	87
	Number of Inter-Experts	10
	Top-K Activation	5
	Load-Balancing Loss Weight	0.001
	Dropout	0.5
**Dynamic Prompt Cross-Attention**	Number of Attention Heads	4
	Head Dimension	32
	Number of Learnable Prompts	8
**CDAN Discriminator**	Domain Adaptation Loss Weight	0.01
	Number of Layers	3

References

[1] Shai Ben-David, John Blitzer, Koby Crammer, and Fernando C Pereira. Analysis of representations for domain adaptation. In *Neural Information Processing Systems*, 2006.

[2] Leo Klarner, Tim G. J. Rudner, Michael Reutlinger, Torsten Schindler, Garrett M. Morris, Charlotte M. Deane, and Yee Whye Teh. Drug discovery under covariate shift with domain-informed prior distributions over functions. In *International Conference on Machine Learning*, 2023.

[3] Muaed Jamal Alomar. Factors affecting the development of adverse drug reactions (review article). *Saudi pharmaceutical journal : SPJ : the official publication of the Saudi Pharmaceutical Society*, 22 2:83–94, 2014.

[4] ML Caballero and Santiago Quirce. Immediate hypersensitivity reactions caused by drug excipients: A literature review. *Journal of investigational allergology & clinical immunology*, 30 2:86–100, 2020.

[5] Mateusz Maciejewski, Eugen Lounkine, Steven Whitebread, Pierre Farmer, William DuMouchel, Brian K. Shoichet, and Laszlo Urban. Reverse translation of adverse event reports paves the way for de-risking preclinical off-targets. *eLife*, 6, 2017.

[6] Craig Knox, Michael Wilson, Christen M. Klinger, Mark N. Franklin, Eponine Oler, Alex Wilson, Allison Pon, Jordan Cox, Na Eun Lucy Chin, Seth A Strawbridge, Marysol Garcia-Patino, Ray Kruger, Aadhavya Sivakumaran, Selena Sanford, Rahil Doshi, Nitya Khetarpal, Omolola Temitope Fatokun, Daphnee Doucet, Ashley Zubkowski, Dorsa Yahya Rayat, Hayley Jackson, Karxena Harford, Afia Anjum, M O Rzayev Zakir, Fei Wang, Siyang Tian, Brian L. Lee, Jaanus Liigand, Harrison Peters, Ruo Qi Rachel Wang, T Chi Nguyen, Denise Rodrigues So, Matthew Sharp, Rodolfo de Tarso Da Silva, Cyrella Gabriel, Joshua Scantlebury, Marissa Jasinski, David Alan Ackerman, Timothy Jewison, Tanvir Sajed, Vasuk Gautam, and David S Wishart. Drugbank 6.0: the drugbank knowledgebase for 2024. *Nucleic Acids Research*, 52:D1265 – D1275, 2023.

[7] Federico Carbone, Atbin Djamshidian, Klaus Seppi, and Werner Poewe. Apomorphine for parkinson's disease: Efficacy and safety of current and new formulations. *CNS Drugs*, 33:905 – 918, 2019.

[8] Jpw Heaton. Apomorphine: an update of clinical trial results. *International Journal of Impotence Research*, 12:S67–S73, 2000.

[9] Maurizio Koch, Angelo Dezi, Fabio Ferrario, and Lucio Capurso. Prevention of nonsteroidal anti-inflammatory drug—induced gastrointestinal mucosal injury: A meta-analysis of randomized controlled clinical trials. *JAMA Internal Medicine*, 156:2321–2332, 1996.

[10] Kuhn M, Letunic I, Jensen LJ, Bork P. Sider: Side effect resource. Database, 2016. drug ID 42955.
